# The portfolio dietary pattern and risk of cardiovascular disease mortality during 1988–2019 in US adults: a prospective cohort study

**DOI:** 10.1186/s12916-025-04067-1

**Published:** 2025-05-21

**Authors:** Meaghan E. Kavanagh, Andreea Zurbau, Andrea J. Glenn, Julianah O. Oguntala, Robert G. Josse, Vasanti S. Malik, Laura Chiavaroli, Simin Liu, Cyril W. C. Kendall, David J. A. Jenkins, John L. Sievenpiper

**Affiliations:** 1 Department of Nutritional Sciences, Temerty Faculty of Medicine, University of Toronto, Toronto, ON Canada; 2 Toronto 3D Knowledge Synthesis and Clinical Trials Unit, Clinical Nutrition and Risk Factor Modification Center, St. Michael’s Hospital, Unity Health Toronto, Toronto, ON Canada; 3 Department of Nutrition, Harvard T.H. Chan School of Public Health, Boston, MA USA; 4 Department of Nutrition and Food Studies, New York University, New York, NY USA; 5 Department of Medicine, Temerty Faculty of Medicine, University of Toronto, Toronto, ON Canada; 6 Division of Endocrinology and Metabolism, St. Michael’s Hospital, Unity Health Toronto, Toronto, ON Canada; 7 Joannah & Brian Lawson Centre for Child Nutrition, Temerty Faculty of Medicine, University of Toronto, Toronto, ON Canada; 8 Li Ka Shing Knowledge Institute, St. Michael’s Hospital, Unity Health Toronto, Toronto, ON Canada; 9 Department of Epidemiology & Biostatistics, Joe C. Wen School of Population and Public Health, University of California, Irvine, CA USA; 10 Center for Global Cardiometabolic Health & Nutrition, University of California, Irvine, CA USA; 11 Division of Cardiology, Department of Medicine, UC Irvine School of Medicine, Irvine, CA USA; 12 College of Pharmacy and Nutrition, University of Saskatchewan, Saskatoon, SK Canada

**Keywords:** Dietary pattern, Portfolio diet, Dietary portfolio, Plant-based, Cholesterol reduction, Cardiovascular disease mortality, All-cause mortality, Prospective cohort study

## Abstract

**Background:**

The Portfolio Diet, a dietary pattern of cholesterol-lowering foods, has been shown to reduce cardiovascular disease (CVD) risk factors in clinical trials and lower CVD risk in observational cohorts of mainly white men and women. However, evidence on mortality outcomes in diverse populations is limited.

**Objective:**

To examine the association of the Portfolio dietary pattern with CVD mortality in a racially diverse cohort.

**Methods:**

A total of 14,835 US adults from the National Health and Nutrition Examination Survey, NHANES (1988–1994), were included. Diet was assessed by a 24-h dietary recall which was supplemented with a food frequency questionnaire at baseline using the Portfolio Diet Score (PDS), with positive points for nuts, plant protein, viscous fiber, phytosterols, and plant monounsaturated fatty acid sources, and negative points for foods high in saturated fat and cholesterol (range, 6–30 points). The primary outcome was CVD mortality. Other mortality outcomes included coronary heart disease (CHD), stroke, and all-cause mortality.

**Results:**

During 22 years of follow-up, 2300 CVD deaths, including 1887 CHD deaths, 413 stroke deaths, and 6238 all-cause deaths were documented. Greater adherence was inversely associated with risk factors including blood lipids, glycemia, and inflammation. Treated as a continuous variable, an increase in PDS by 8 points was associated with a 12% (hazard ratio 0.88 [95% confidence intervals:0.78, 0.99]), 14% (0.86 [0.78, 0.96]), and 12% (0.88 [0.82, 0.95]) lower risk of CVD, CHD, and all-cause mortality after adjustments for known CVD risk factors. Comparing the highest to lowest tertiles of the PDS, higher PDS was associated with 16% (0.84 [0.73, 0.98]), 18% (0.82 [0.72, 0.95]) and 14% (0.86 [0.78, 0.96]) lower risk of CVD, CHD, and all-cause mortality, respectively. As part of exploratory analyses, an interaction between PDS and race/ethnicity was observed, emphasizing the necessity of future research involving underserved groups.

**Conclusions:**

Among a national cohort of racially diverse adults in the US, greater adherence to the Portfolio dietary pattern was inversely and prospectively associated with CVD, CHD, and all-cause mortality.

**Supplementary Information:**

The online version contains supplementary material available at 10.1186/s12916-025-04067-1.

## Background

Cardiovascular disease (CVD) persists as a leading cause of death in the United States (US) [1], despite effective pharmacotherapies. Direct costs of CVD range from 11 to 15% of all health care expenditures for Group 20 (G- 20 +) countries [2], posing a looming threat to healthcare systems [3]. Dietary modification has been consistently established as front-line therapy for the management of dyslipidemia [4] and as a leading strategy for primary and secondary prevention of CVD [5, 6]. Nearly half of US adults have poor diet quality [7], underscoring an opportunity to enhance public health.

Recognizing the potential for even modest shifts in the population’s diet to reduce CVD prevalence, it is important to investigate beneficial dietary patterns. Among the new dietary approaches recognized for CVD prevention, the Portfolio dietary pattern has emerged as a promising strategy with high-quality evidence for the management of dyslipidemia [8]. This pattern strategically combines well-established cholesterol-lowering plant foods, mirroring pharmacotherapies with demonstrated efficacy for atherosclerotic cardiovascular disease (ASCVD), by targeting plasma low-density lipoprotein cholesterol (LDL-C) levels. Foundational research found that the Portfolio dietary pattern lowered LDL-C by ~ 30% and was analogous to a first-generation statin [9]. The targeting of LDL-C is underpinned by multiple lines of evidence [10] which collectively have established that LDL-C is causal in the development of ASCVD [11]. Beyond LDL-C, the Portfolio dietary pattern has demonstrated clinically meaningful benefits on other established targets including non-high-density lipoprotein-cholesterol (non-HDL-C), apolipoprotein B (apoB), triglycerides, blood pressure, C-reactive protein (CRP), leading to a reduced 10-year CVD risk [12].

Despite these benefits on cardiovascular parameters, it remains unclear whether adherence to the Portfolio dietary pattern translates to reductions in cardiovascular outcomes, especially cardiovascular mortality. Analysis of the National Health and Nutrition Examination Survey (NHANES) III (1988–1994) provides an opportunity to investigate this association among a national cohort of racially diverse adults in the US. Previously, the Portfolio dietary pattern has been associated with a lower CVD risk in the Women’s Health Initiative (WHI) [13] and the Nurses’ Health Studies and Physicians Follow-up Study [14]. However, these findings have been in predominately White adults (84 and 97%, respectively). This study aims to examine the association of the Portfolio dietary pattern with CVD mortality among a national cohort of racially diverse adults in the US.

## Methods

### Study population

NHANES is a nationally representative, cross-sectional, multistage probability survey of the civilian, noninstitutionalized US population with deliberate oversampling of non-Hispanic Black populations, Mexican American populations, and persons over the age of 60 years. NHANES III comprises two 3-year phases conducted from 1988–1994 [15]. NHANES III received institutional review board approval from the National Center for Health Statistics Research Ethics Review Board (USA CDC, 2015) and required the provision of written informed consent.

NHANES (1988–1994) was selected because of its longer follow-up (more than 10 years of additional follow-up than that of NHANES post 1999). The longer follow-up was necessary to accrue sufficient CVD mortality events. The study protocol was prespecified and approved (ID:2202) by Centers for Disease Control and Prevention (CDC). Figure S1 displays the study population selection. The study included nonpregnant adults aged 20 years or older (*n* = 18,537) with a complete first-day 24-h dietary recall who were eligible for mortality follow-up (*n* = 15,687), excluding those with BMI below 18.5 kg/m^2^ and with missing covariate values, resulting in 14,835 adults.

### Estimate of the portfolio dietary pattern

Dietary interviews were administered in English and Spanish in the mobile examination center (MEC). A single 24-h dietary recall was used to determine dietary intake together with a food frequency questionnaire (FFQ). The Portfolio dietary pattern is comprised of many episodically consumed foods (nuts, lentils, beans, soy foods, oat products, barley, etc.). As a single 24-h recall is subject to random within-person error, it can miss these episodically consumed foods (non-consumers) leading to an overestimate of never-consumers [16]. While FFQs are considered good instruments for comparing intakes across groups, they are prone to random and systematic errors [17]. Thus, it has been suggested to combine 24-h recalls with FFQs, as the FFQ data may improve the estimation of the best conditional expectation based on 24-h recalls [18]. The non-quantitative FFQ included within NHANES III used a 1-month reference period. We used the FFQ to disassociate never-consumers from non-consumers for all categories, except phytosterols. Phytosterols were omitted because the FFQ was not designed to produce population nutrient intake estimates.

To assess adherence with the Portfolio dietary pattern, we used the validated Portfolio Diet Score (PDS), ranging from 6 to 30 points [19]. Additional file 1:Table S1 shows the scoring criteria for each Portfolio dietary pattern category. Foods from the 24-h recall were categorized by the pattern’s components. Servings were calculated using the USDA’s Food and Nutrient Database for Dietary Studies (FNDDS) portions dataset [20]. Intake was assessed as servings/day of food components from the 24-h recall, except for phytosterols. Phytosterols intake was instead based on all 24-h recall items to derive total intake (mg/day). Phytosterol content of foods was estimated from 3 databases [21–23], as done previously [19].

### Outcomes

The primary outcome was CVD mortality, other outcomes included CHD, stroke, and all-cause mortality. Mortality data were obtained from National Death Index deaths certificate records until 31 December 2019 [24], described in Additional file 1: Appendix 1 [24].

For biomarkers, baseline blood samples were collected from participants at the MEC. LDL-C was only calculated in persons who had triglycerides below 800 mg/dL (*n* = 13,974) in accordance with the NIH Eq. [25]. Non–HDL-C was calculated by subtracting HDL-C from total cholesterol.

### Analyses

According to analytical guidelines [26], all analyses were weighted using MEC final examination weights, in addition to the strata and their primary sampling units (PSU) codes, to account for the complex sampling design. Characteristics were expressed as weighted means ± standard error (SE) for continuous variables and were compared using *t*-tests. Categorical variables were expressed as weighted percentages ± SE and were compared using the Wald chi-squared test. Person years of follow-up were calculated from the date of the examination until the earliest time of death by any cause or the end of follow-up. Statistical analyses were performed using SAS version 9.4 (SAS Institute Inc.). All tests were two-tailed, and *P* < 0.05 were prespecified to be considered significant. However, to algin with recommendations from the American Statistical Association, associated estimates and uncertainties were interpreted in context rather than relying on a strict *P*-value threshold [27]. Additional file 1:Figure S2 presents a direct acyclic graph constructed from literature [28–31] and expert knowledge used to identify potential confounders related to CVD [32], created using the DAGitty web application [33]. Covariate assessments are described in Additional file 1:Appendix 2 with adjustment for baseline sociodemographic, medical history, family history, lifestyle, and dietary characteristics. The proportional hazards assumptions were assessed, and time dependency was checked. Weighted Cox proportional hazards regression models (SURVEYPHREG) were used to estimate the hazard ratios (HRs) and 95% confidence intervals (CI) for mortality outcomes, comparing high with low tertiles of the PDS. Due to the low incidence of the mortality outcomes, hazards were interpreted as risks. To quantify a linear trend, the weighted mean value of the PDS within each tertile was assigned and the exposure was modeled as a continuous variable. The PDS was also assessed continuously per 8 points (33 percentile increments). To better to understand how the hazard may change over time [34, 35], adjusted survival curves were produced by computing model-based survival estimates standardized to the covariate distribution of the study population. To visually and statistically assess possible nonlinear associations, the %RCS_REG SAS macro (Version V1.4 beta) by Loic Desquilbet [36] was used to plot a restricted cubic spline (RCS) function with three knots at the 5 th, 50 th, and 95 th percentiles of the PDS distribution and mortality outcomes using cox-regression, allowing for graphical characterization of nonlinear associations. Procedures for biomarker and sensitivity analyses, mediation analysis of BMI, and stratified analysis including by race/ethnicity are described in Additional file 1:Appendix 3 [27].

## Results

### Population

We included 14,835 adults, among whom non-Hispanic Black adults and Mexican American adults were purposely oversampled (total number [weighted percentage]: 6121 [76%] non-Hispanic White adults, 4162 [11%] non-Hispanic Blacks adults, 3978 [5%] Mexican Americans adults, and 574 [7.5%] other). During a mean follow-up of 22 years, a total of 2300 CVD deaths (including 1887 CHD and 413 stroke deaths) and 6238 all-cause deaths were documented over 326,544 person-years. Table 1 presents participants characteristics by tertiles of PDS. Participants with a higher PDS were older, reported a higher calorie intake, and were less likely to be current smokers. Additional file 1:Table S2 presents the mean servings for each of the PDS components by tertiles.

**Table 1:** Baseline characteristics of US adult participants in the National Health and Nutrition Examination Survey 1988–1994 by tertiles of the Portfolio Diet Score, weighted prevalence and means (SE)

**Characteristic**		**T1 (low)**	**T2 (medium)**	**T3 (high)**
**No**		4832	4251	5752
**Dietary score, mean (SE)**		14.0 (0.04)	18.0 (0.02)	22.2 (0.04)
**Age, mean (SE), year**		43.1 (0.6)	44.5 (0.6)	47.2 (0.6)
**Sex, % (SE)**				
	Male	47.6 (0.9)	49.0 (1.3)	49.8 (0.9)
	Female	52.4 (0.9)	51.0 (1.5)	50.2 (0.9)
**Race/ethnicity, % (SE)**				
	Non-Hispanic White	73.5 (1.4)	76.1 (1.6)	79.3 (1.3)
	Non-Hispanic Black	14.5 (0.8)	10.4 (0.7)	8.4 (0.66)
	Mexican American	3.9 (0.3)	5.8 (0.5)	5.4 (0.5)
	Other^a^	8.1 (1.0)	7.7 (1.1)	6.9 (0.88)
**Education, mean (SE), year**		12.3 (0.1)	12.5 (0.1)	13.1 (0.1)
**Smoking status, % (SE)**				
	Current	36.4 (1.4)	30.7 (1.0)	19.7 (1.1)
	Former	20.7 (0.9)	25.3 (0.9)	31.0 (1.0)
	Never	42.8 (1.4)	44.0 (0.9)	49.3 (1.2)
**Alcohol intake, % (SE)**				
	None	45.1 (1.5)	47.0 (1.7)	47.0 (1.8)
	< 3 drinks/week	26.0 (1.1)	26.7(1.1)	25.8 (1.1)
	≥ 3 drinks/week	28.9 (1.4)	26.4 (1.5)	27.2 (1.3)
**Physical activity, % (SE)**				
	Most active (≥ 5 times/week)	34.0 (1.3)	40.9 (1.9)	48.4 (1.4)
	Moderate (< 5 times/week)	47.7 (1.3)	44.2 (1.8)	39.6 (1.1)
	Inactive (0 times/week)	18.3 (1.1)	14.9 (0.9)	12.0 (1.0)
**Poverty Income Ratio, % (SE)**				
	Low (< 1.3)	25.7 (1.14)	24.1 (1.31)	19.8 (1.2)
	Middle (1.3 to 3.5)	45.6 (1.2)	43.7 (1.7)	40.1 (1.3)
	High (> 3.5)	28.7 (1.2)	32.2 (1.8)	40.1 (1.6)
**Marital status, % (SE)**				
	Married	62.1 (1.2)	67.0 (1.6)	70.0 (1.0)
	Widowed, divorced, separated	20.2 (1.0)	18.7 (0.9)	15.4 (0.8)
	Never married	17.7 (1.0)	14.4 (1.3)	14.6 (0.9)
**Family history of early CVD, % (SE)**		12.2 (0.7)	11.3 (0.7)	10.1 (0.6)
**Self-reported hypertension, % (SE)**		23.1 (1.0)	24.1 (1.0)	26.0 (1.0)
**Self-reported hypercholesteremia**^**b**^**, % (SE)**		35.1 (1.6)	34.9 (1.7)	35.6 (1.0)
**Self-reported diabetes, % (SE)**		5.0 (0.4)	5.6 (0.4)	6.0 (0.4)
**History of cancer**^c^**, % (SE)**		2.8 (0.4)	4.0 (0.5)	4.6 (0.3)
**BMI, mean (SE), kg/m**^**2**^		27.0 (0.2)	26.9 (0.2)	26.4 (0.1)
**Systolic blood pressure, mean (SE), mm Hg**		125.3 (2.0)	123.8 (1.2)	124.9 (0.8)
**Diastolic blood pressure, mean (SE), mm Hg**		77.4 (2.2)	75.9 (1.2)	75.9 (0.5)
**HbA1c, mean (SE), %**		5.4 (0.03)	5.4 (0.03)	5.4 (0.03)
**Non-HDL-C**^**b**^**, mean (SE), mg/dL**		154.5 (1.2)	154.4 (1.2)	154.6 (1.1)
**LDL-C**^**b**^**, mean (SE), mg/dL**		129.0 (1.2)	128.9 (1.0)	128.7 (0.8)
**CRP**^**b**^**, mean (SE), mg/dL**		0.44 (0.01)	0.42 (0.02)	0.38 (0.01)
**Total energy intake, mean (SE), kcal/d**		2060.6 (21.9)	2147.1 (30.7)	2331.1 (34.7)
**Dietary sodium, mean (SE), mg**		3427.0 (45.4)	3464.2 (53.4)	3723.1 (68.4)
**HEI, mean (SE)**		58.5 (0.3)	62.7 (0.3)	68.7 (0.3)

T1 (low), T2 (mid), and T3 (highest) tertiles of adherence to the Portfolio dietary pattern measured using the Portfolio Diet Score (PDS)

LDL-C was only calculated in persons who had triglycerides below 800 mg/dL (*n* = 13,974) in accordance with the NIH Eq. [25]

To convert non-HDL-C and LDL-C from mg/dL to mmol/L divide by 38.67

To convert CRP mg/dL to mg/L multiple by 10

*Abbreviations*: *BMI* body mass index, *CRP* C-reactive protein, *CVD* cardiovascular disease, *HEI* Healthy Eating Index, *HDL-C *high density lipoprotein cholesterol,* HbA1c *hemoglobin A1c, *LDL-C* low-density lipoprotein cholesterol, *T2D *Type- 2 diabetes, *T1* first tertile, *T2* second tertile, *T3* third tertile, *SE* standard error

^a^Other race/ethnicity includes all other Hispanics regardless of race (such as Other Latin American/Spanish ancestry or national origin) and all other non-Hispanic adults from racial groups other than White or Black (i.e., American Indian or Alaskan Native; Native Hawaiian or Pacific Islander; multiple races or ethnicities; or unknown)

^b﻿^More than 5% with missing values (*n* = missing; self-reported hypercholesteremia, *n* = 7104; non-HDL, *n* = 814; LDL-C, *n* = 861; CRP, *n* = 807)

^c﻿^History of cancer excluded skin cancer

### Mortality outcomes

**Fig. 1:**
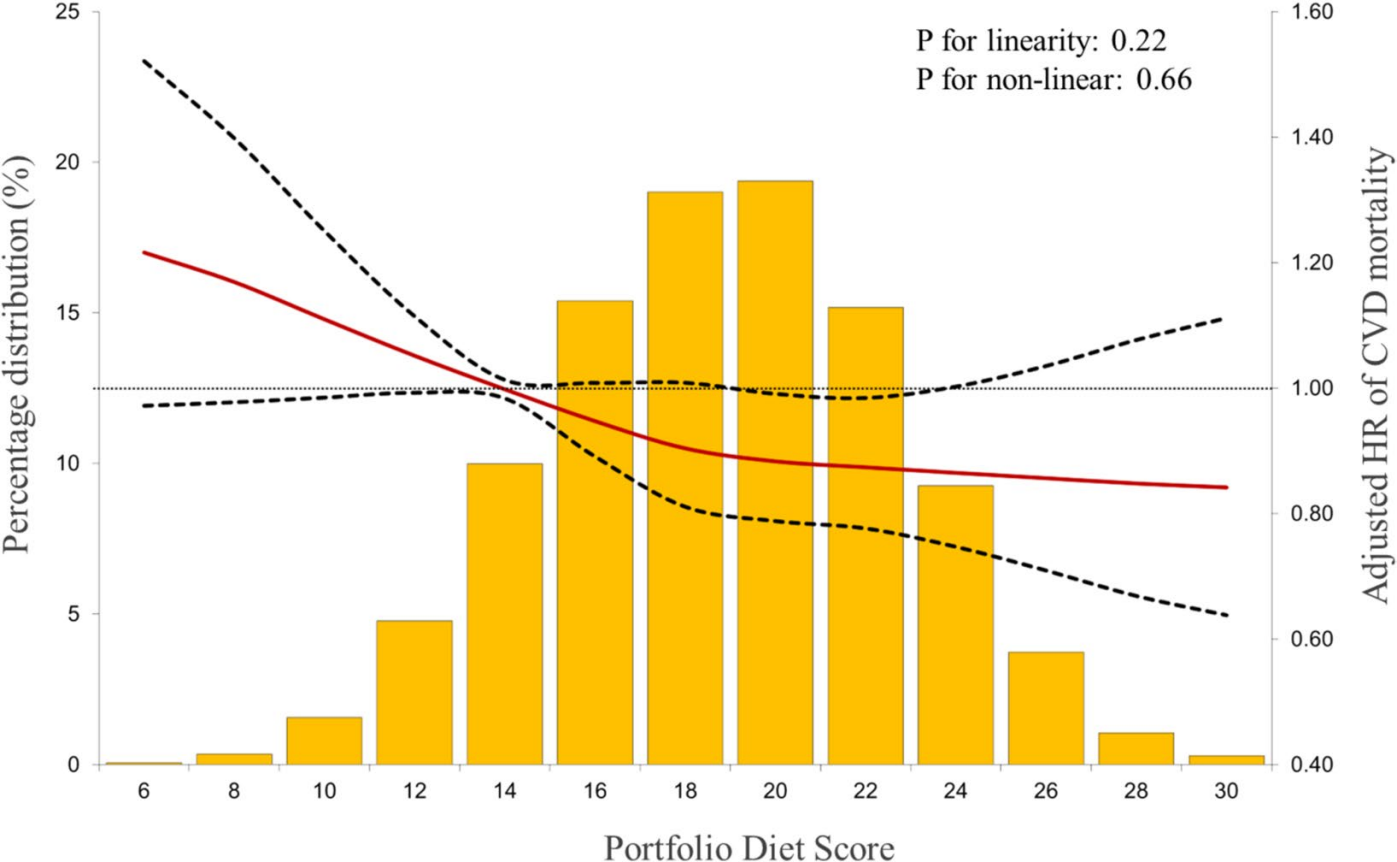
Distributions of Portfolio Diet Score (PDS) and adjusted differences in hazard ratio for primary outcome of CVD mortality* using 13.97 as reference value (midpoint of the lowest tertile of PDS), in US adults: National Health and Nutrition Examination Survey Linked Mortality Files (1988–2019). Abbreviations: CI, confidence intervals; CVD, cardiovascular disease; HR, hazard ratio. Cox regression models were used to estimate the adjusted differences in HR of CVD^*^ and corresponding 95% CI. HRs were coded using restricted cubic spline (RCS) function with three knots located at the 5th, 50th, and 95th percentiles of the PDS distribution (reference value = 13.9, the mean PDS of tertile 1). Solid red line represents HR and dotted lines represent 95% CIs. Estimates were adjusted by age, sex, and race/ethnicity, educational attainment, smoking status, Poverty Income Ratio, marital status, physical activity, alcohol consumption, family history of CVD, self-reported cancer (other than skin), self-reported type-2 diabetes, self-reported hypertension, energy intake, and sodium. *CVD mortality defined as deaths identified as Disease of heart (ICD codes: I00-I09, I11, I13, I20-I51) and Cerebrovascular disease (ICD codes I60-I69)

Table 2 shows the adjusted HR for the primary outcome of CVD mortality in all participants (*n*=14,835) for an 8-point increase in PDS, according to PDS by tertiles, and *P*-value for trend. Figure 1 shows the distribution of the PDS and adjusted differences in HR for the primary outcome of CVD mortality using the mean of tertile 1 (13.9 points) as the reference value. For all participants, an 8-point increase in PDS was associated with a lower risk of CVD mortality (HR 0.88; 95% CI 0.78, 0.99). When comparing the highest to lowest tertiles of intake, a higher PDS was associated with a lower risk of CVD mortality (HR 0.84; 95% CI 0.73, 0.98; *P*_trend_ = 0.031), after multivariable adjustments (Table 2). Additional file 1:Table S3 presents adjusted HR for CVD mortality by baseline CVD status. Findings remained consistent when those with CVD at baseline were removed and no interaction by baseline CVD status was found. For secondary prevention in those who reported CVD at baseline (*n* = 1,249), no association with PDS was observed for CVD mortality.

**Table 2:** Adjusted hazard ratio for mortality outcomes according to tertiles of the Portfolio Diet Score: National Health and Nutrition Examination Survey Linked Mortality Files (1988–2019)

Characteristic	T1 (low)	T2 (medium)	T3 (high)	*P* value for trend	Per 8 points, HR (95% CI)
**CVD mortality**^a^					
Cases	731	652	917		
No. of participants	4832	4251	5752		
Person years	106,397	94,328	125,818		
Adjusted for age, sex, race/ethnicity, HR (95% CI)	1 [Ref]	0.83 (0.73, 0.96)	0.74 (0.64, 0.85)	< 0.001	0.76 (0.67, 0.87)
Model 2, HR (95% CI)	1 [Ref]	0.85 (0.74, 0.99)	0.82 (0.71, 0.95)	0.011	0.86 (0.76, 0.98)
Model 3, HR (95% CI)	1 [Ref]	0.86 (0.74, 0.99)	0.84 (0.73, 0.98)	0.031	0.88 (0.78, 0.99)
**CHD mortality**^b^					
Cases	602	543	742		
No. of participants	4832	4251	5752		
Person years	106,397	94,328	125,818		
Adjusted for age, sex, race/ethnicity, HR (95% CI)	1 [Ref]	0.82 (0.70, 0.95)	0.71 (0.62, 0.82)	< 0.001	0.73 (0.64, 0.83)
Model 2, HR (95% CI)	1 [Ref]	0.83 (0.71, 0.98)	0.81 (0.70, 0.92)	< 0.001	0.84 (0.74, 0.95)
Model 3, HR (95% CI)	1 [Ref]	0.84 (0.72, 0.99)	0.82 (0.72, 0.95)	0.010	0.86 (0.76, 0.96)
**Stroke mortality**^c^					
Cases	129	109	175		
No. of participants	4832	4251	5752		
Person years	106,397	94,328	125,818		
Adjusted for age, sex, race/ethnicity, HR (95% CI)	1 [Ref]	0.93 (0.61, 1.43)	0.88 (0.58, 1.34)	0.56	0.95 (0.70, 1.29)
Model 2, HR (95% CI)	1 [Ref]	0.94 (0.54, 1.47)	0.91 (0.59, 1.41)	0.67	0.99 (0.72, 1.37)
Model 3, HR (95% CI)	1 [Ref]	0.94 (0.60, 1.48)	0.95 (0.59, 1.48)	0.84	1.03 (0.75, 1.44)
**All-cause mortality**					
Cases	2021	1753	2464		
Participants	4832	4251	5752		
Person years	106,397	94,328	125,818		
Adjusted for age, sex, race/ethnicity, HR (95% CI)	1 [Ref]	0.85 (0.76, 0.95)	0.75 (0.68, 0.83)	<.0001	0.76 (0.70, 0.83)
Model 2, HR (95% CI)	1 [Ref]	0.87 (0.79, 0.96)	0.85 (0.76, 0.95)	0.006	0.86 (0.78, 0.96)
Model 3, HR (95% CI)	1 [Ref]	0.88 (0.80, 0.96)	0.86 (0.78, 0.96)	0.008	0.88 (0.81, 0.95)

T1 (low), T2 (mid), and T3 (highest) tertiles of adherence to the Portfolio dietary pattern measured using the Portfolio Diet Score (PDS)

Model 2: age (continuous), sex (male; female), and race/ethnicity (non-Hispanic White adults; non-Hispanic Black adults; Mexican American adults; other), educational attainment (< 12; 12; > 12 years), smoking status (never; former; current), Poverty Income Ratio (low [< 1.3]; middle [1.3 to 3.5]; high [> 3.5]),), marital status (married/living with partner; divorced/widowed/separated; never married), physical activity (0; 0 < to 5; ≥ 5 times/week of moderate- to vigorous-intensity activities), alcohol consumption (0; 0 < to 3; ≥ 3 drinks/week), family history of CVD (yes; no), self-reported cancer other than skin (yes; no), self-reported type- 2 diabetes (yes; no), and self-reported hypertension (yes; no)

Model 3: age (continuous), sex (male; female), and race/ethnicity (non-Hispanic White adults; non-Hispanic Black adults; Mexican American adults; other), educational attainment (< 12; 12; > 12 years), smoking status (never; former; current), Poverty Income Ratio (low [< 1.3]; middle [1.3 to 3.5]; high [> 3.5]),), marital status (married/living with partner; divorced/widowed/separated; never married), physical activity (0; 0 < to > 5; ≥ 5 times/week of moderate- to vigorous-intensity activities), alcohol consumption (0; 0 < to > 3; ≥ 3 drinks/week), family history of CVD (yes; no), self-reported cancer other than skin (yes; no), self-reported type- 2 diabetes (yes; no), and self-reported hypertension (yes; no), energy intake (continuous), and sodium (continuous)

*Abbreviations*: *CI* confidence intervals, *CHD* coronary heart disease, *CVD* cardiovascular disease, *HR* hazard ratio, *T1* first tertile, *T2* second tertile, *T3* third tertile, *SE* Standard Error

^a^CVD mortality defined as deaths identified as Disease of heart (ICD codes: I00-I09, I11, I13, I20-I51) and Cerebrovascular disease (ICD codes I60-I69)

^b^CHD mortality defined as deaths identified as Disease of heart (ICD codes: I00-I09, I11, I13, I20-I51)

^c^Stroke mortality defined as deaths identified as Cerebrovascular disease (ICD codes I60-I69)

Table 2 shows the adjusted HR of CHD, stroke, and all-cause mortality for all participants for an 8-point increase in PDS, according to PDS by tertiles, and *P*-value for trend. For all participants, an 8-point increase in PDS was associated with a lower risk of CHD mortality (0.86; 95% CI 0.78, 0.96) and all-cause mortality (0.88; 95% CI: 0.82, 0.95) after multivariable adjustments. No association was observed for stroke mortality (1.03; 95% CI: 0.75, 1.44). When comparing the highest to lowest tertiles of intake, a higher PDS was associated with a lower risk of CHD mortality (0.82; 95% CI 0.72, 0.95; *P*_trend_ < 0.01), and all-cause mortality (0.86; 95% CI 0.78, 0.96; *P*_trend_ = 0.008). No association was observed for stroke mortality (0.95; 95% CI 0.59, 1.48; *P*_trend_ = 0.84). Additional file 1:Tables S4 - 6 present adjusted HR for CHD, stroke, and all-cause mortality by baseline CVD status. Again, no interaction by baseline CVD status was observed.

Additional file 1: Figure S3 presents the survival curves for all outcomes over the duration of the follow-up period. Additional file 1: Figure S4 presents adjusted differences in HR by PDS distribution for all outcomes with tests for linearity and non-linearity. There was no strong evidence of linear or non-linear associations for any outcome.

### Exploratory subgroup analyses

Additional file 1: Figure S5 - 8 presents the stratified analysis. Interaction terms between PDS and each subgroup showed little evidence of interaction, except for race/ethnicity in relation to CVD, and CHD mortality (*P* < 0.01; Figure S5 and S6). There was no strong evidence of interaction for stroke except for physical activity (*P* = 0.029; Figure S7), and no strong evidence of interaction for all-cause mortality (*P* = 0.18; Figure S8). Table 3 presents the subgroup analysis estimates for race/ethnicity for CVD and CHD mortality. The direction of the association with CVD and CHD mortality was the same for both race/ethnicity groups, with a pattern of greater reductions for CVD in the non-White group (0.57; 95% CI 0.45, 0.72) compared to the White group (0.92; 95% CI 0.77, 1.11) with a *P* < 0.01 for interaction, and for CHD in the non-White group (0.53; 95% CI 0.41, 0.69) compared to the White group (0.91; 95% CI 0.77, 1.07) with a *P* < 0.01 for interaction (Additional file 1:Figure S5 and S6). Additional file 1:Table S10 presents the subgroup analysis estimates by the original four race/ethnicity groupings used in NHANES III (non-Hispanic White; non-Hispanic Black; Mexican American; other).

**Table 3:** Exploratory subgroup analysis estimates for Portfolio Diet Score and race/ethnicity with adjusted hazard ratio for race/ethnicity in relation to CVD mortality and CHD mortality: National Health and Nutrition Examination Survey Linked Mortality Files (1988–2019)

**Race/ethnicity**	**Participants no**	**Events no**	**Adjusted HR (95% CI)**	***P* value** **for interaction**^‡^
**CVD mortality**				0.008
White*	4440	936	0.92 (0.77, 1.11)	
Non-White†	6144	712	0.57 (0.45, 0.72)	
**CHD mortality**				0.004
White*	4440	779	0.91 (0.77, 1.07)	
Non-White†	6144	565	0.53 (0.41, 0.69)	

Comparing Tertile 3 (highest) with Tertile 1 (lowest) of Portfolio Diet Score by Selected Characteristics Among US Adults 20 Years or Older: National Health and Nutrition Examination Survey (NHANES) Linked Mortality Files, 1988–2019. Estimates were adjusted by age, sex, and race/ethnicity, educational attainment, smoking status, Poverty Income Ratio, marital status, physical activity, alcohol consumption, family history of CVD, self-reported cancer (other than skin), self-reported type- 2 diabetes, self-reported hypertension, energy intake, and sodium

For all subgroups see Figures S5 - 8

*Abbreviations*: *CI* confidence intervals, *CHD* coronary heart disease, *CVD* cardiovascular disease, *HR* hazard ratio

^*^White includes reported non-Hispanic White race/ethnicity

^†^non-White includes (non-Hispanic Black adults, Mexican American adults, and Other race/ethnicity). The Other race/ethnicity includes all other Hispanics regardless of race (such as Other Latin American/Spanish ancestry or national origin) and all other non-Hispanic adults from racial groups other than White or Black (i.e., American Indian or Alaskan Native; Native Hawaiian or Pacific Islander; multiple races or ethnicities; or unknown). Consolidated owing to small numbers

^‡^*P* value is for interaction across subgroups for all tertiles

### Sensitivity analyses

Additional file 1: Table S7 shows the Spearman correlations between the PDS and Healthy Eating Index (HEI) (*r* = 0.35). Additional file 1:Table S8 presents the BMI-adjusted analysis. Adjustment for BMI as a potential mediator slightly attenuated the association between tertiles by 1–2% for CVD, CHD, and all-cause mortality though the overall associations remained consistent.

### Biomarkers

Table 4 shows the linear regression analysis between PDS and CVD biomarkers in a cross-sectional analysis with results presented as beta-coefficients (β) for a 1-point change in PDS in addition to an 8-point change. The PDS was inversely associated with established lipid targets [8], LDL-C (*β* − 0.37 mg/dL, SE 0.13, *P* = 0.006), and non-HDL-C (*β* − 0.36 mg/dL, SE 0.15,* P* = 0.016). Additionally, PDS was inversely associated with total cholesterol (*β* − 0.41 mg/dL, SE 0.14, *P* = 0.007), CRP (*β* − 0.01%, SE:0.002, *P* < 0.001), and HbA1c (*β* − 0.01%, SE 0.002, *P* < 0.001), while no associations were found for triglycerides, HDL-C, or blood pressure.

**Table 4:** Association between the Portfolio Diet Score and baseline cardiometabolic biomarkers, a cross-sectional analysis in NHANES 1988–1994

**Biomarker**	**B coefficient (SE)**	**Associated change** **per 8-point increase in PDS**	***P* value** **linear regression**
**LDL-C, mg/dL**	− 0.37 (0.1)	− 2.93 mg/dl	0.006
**Non-HDL-C, mg/dL**	− 0.36 (0.2)	− 2.90 mg/dl	0.016
**Total cholesterol, mg/dL**	− 0.41 (0.1)	− 3.3 mg/dl	0.007
**HDL-C, mg/dL**	0.009 (0.1)	0.08 mg/dl	0.85
**Triglycerides, mg/dL**^**a**^	− 0.002 (0.002)	− 0.56%	0.26
**HbA1c, %**	− 0.01 (0.002)	− 0.06%	< 0.001
**CRP, mg/dL**^**a**^	− 0.01 (0.002)	− 6.05%	< 0.001
**Systolic BP, mm Hg**^**a**^	− 0.001 (0.001)	− 0.75%	0.13
**Diastolic BP, mm Hg**^**a**^	− 0.0003 (0.001)	− 0.23%	0.65

Linear regression analyses were performed in a cross-sectional analysis between the PDS and clinical biomarkers of CVD risk (lipids, glucose control, blood pressure, and inflammation). Prior to analysis, univariate statistics of all dependent variables were assessed and those which were extremely non-normal were log transformed to an approximate normal distribution

All estimates were adjusted by age, sex, and race/ethnicity, educational attainment, smoking status, Poverty Income Ratio, marital status, physical activity, alcohol consumption, family history of CVD, self-reported cancer (other than skin), self-reported type- 2 diabetes, self-reported hypertension, energy intake, and sodium

*P* values were based on weighted data and are presented for linear relationships

LDL-C was only calculated in persons who had triglycerides below 800 mg/dL (*n* = 13,974) in accordance with the NIH Eq. [25]

To convert total cholesterol, HDL-, LDL-C, and non-HDL-C from mg/dL to mmol/L divide by 38.67

To convert triglycerides from mg/dL to mmol/L divide by 88.57

To convert CRP mg/dL to mg/L multiple by 10

*Abbreviations*: *BP* blood pressure, *CRP* C-reactive protein, *HDL-C* high density lipoprotein cholesterol, *HbA1c* hemoglobin A1c, *LDL-C* low density lipoprotein cholesterol, *NHANES* National Health and Nutrition Examination Survey, SE standard error

^a^Log transformed

### Individual food components

Additional file 1: Table S9 presents the associations of the individual Portfolio dietary pattern components and CVD mortality using RCS with the reference value for each component as the mean servings of T1 (i.e., 0.12 servings for nuts). When assessed individually, there were no associations.

## Discussion

We have conducted the first cohort study in representative sample of US adults examining the association of the Portfolio dietary pattern on CVD mortality using dietary data from 24-h recalls and FFQs (*n* = 14,835 with 2300 CVD deaths [including 1887 CHD deaths and, 413 stroke deaths] and 6238 all-cause deaths). Over 22 years of follow-up, greater adherence to the Portfolio dietary pattern was associated with a lower risk of CVD mortality. An increase in PDS by 8 points was associated with a 12, 14, and 12% lower risk of CVD, CHD, and all-cause mortality, after adjustments for known CVD risk factors. Similar findings were also observed when comparing the highest to lowest tertiles, where those with higher adherence to the Portfolio dietary pattern had an associated 16% lower risk of CVD mortality. We also observed an associated 18 and 14% lower risk for CHD and all-cause mortality, respectively, but not for stroke mortality.

Furthermore, in cross-sectional analyses, greater adherence to the Portfolio dietary pattern was inversely associated with LDL-C and non-HDL-C, which are established targets for CVD management [8] and can be considered objective biomarkers of exposure/adherence to the Portfolio Diet, given that the diet was designed to target LDL-C directly [19]. Beyond lipids, an inverse association was seen with other established cardiometabolic risk factors (CRP and HbA1c), aligning with the mortality findings and emphasizing the robustness of our observations.

As NHANES III is a racially diverse sample of US adults with deliberate over-sampling of non-Hispanic Black adults and Mexican American adults, populations previously not included in Portfolio Diet research, these findings provide comprehensive insights into the Portfolio dietary pattern’s associated impact on CVD mortality risk. In our exploratory stratified analysis, an interaction by race/ethnicity for CVD and CHD mortality was observed. The direction of the association was the same for both race/ethnicity groups but there was a pattern of greater associated reductions in the non-White group. When we examined whether the association between the Portfolio dietary pattern and CVD mortality differed by social economic status (SES), including level of education and Poverty Index Ratio, no interactions were observed [37]. While surprising, this finding is similar to past work in NHANES where no strong evidence of interaction between SES and health outcomes was observed [38]. These observed interactions should be interpreted as hypothesis generating for future investigation, allowing for exploration of possible health disparities that may exist between a cholesterol lowering dietary pattern (i.e., the Portfolio dietary pattern) and CVD. A recent National Academy of Sciences report has cautioned use of race and ethnicity in analyses and recommends providing scientific rationale for exploring differences by race rather than genetic, environmental, economic, or other factors [37]. As genetic risk scores were not available and neither were all measures of social inequalities captured, our intension for exploring race/ethnicity categories was to emphasize possible genetic and social inequities, and to highlight the necessity of future research involving underserved groups. An example of a suggested mechanism is that elevated Lipoprotein(a) (Lp(a)) is genetically determined and is present disproportionately in Black populations, relative to White populations [39], with levels being threefold higher in Black than White individuals [40]; however, Lp(a) was only captured in a limited number of participants between 1991 and 1994.

Our work is the first to look at the Portfolio dietary pattern in NHANES III. Previous work with NHANES III has found associated health benefits with other dietary patterns, including the Mediterranean diet [41], the DASH diet [42, 43], and HEI [43]. Despite some common foods with these diets, our correlation analysis of the Portfolio dietary pattern with HEI, along with prior analyses of the DASH and Mediterranean diets, indicates these dietary patterns are not strongly correlated with the Portfolio Diet, as assessed by the PDS [14]. This finding underscores that multiple dietary patterns can be recommended for cardiovascular disease prevention, allowing for catering to individual preferences.

Our findings align with previous findings on the Portfolio dietary pattern. The pattern has been inversely associated with CVD [13, 14], type 2 diabetes [44], and cardiometabolic risk factors in trials and cohorts [12, 45]. Our novel analysis using 24-h recalls provided a high level of precision in coding foods. This approach allowed the separation of foods grouped together in FFQs, offering insights into food preparation methods (e.g., addition of lard in cooking beans) and distinguishing between reduced and full-fat dairy foods which can differ in their saturated fat content. The interaction by ethnicity for the Portfolio dietary pattern and risk of CVD and CHD has not been observed in past analyses [13, 14]. However, these cohorts were composed of predominately White populations (≥ 84%). We did not find an association with stroke mortality, possibly due to the limited number of events. Recently, an analysis from 3 large prospective cohort studies which had a larger sample size found a higher PDS was associated with a lower risk of stroke events, in addition to CVD and CHD events [14].

The consumption of nuts, plant protein, viscous fiber, phytosterols, and MUFAs are low in the US population. These findings emphasize the importance of encouraging consumption of these foods through dietary guidance, as even small increases in population intake may have broad cardiovascular benefits. When comparing the mean intake of each of the categories for the Portfolio Diet to targets used in randomized controlled trials [9, 46], participants in the T1 (low), T2 (mid), and T3 (highest) would be considered ~ 6.7%, 10.1 and 18.8% adherent to the Portfolio Diet, respectively. Therefore, only partial adoption of the Portfolio Diet (difference between T1 and T3 of ~ 12%) may lead to a 16% lower risk of CVD mortality. To translate this finding into food-based servings, those wishing to reach the same adherence as those in the highest tertile (18.8%) would need to eat a combination of five of the following examples: roughly 1 oz of nuts or 2 tablespoons of peanut butter, ½ cup of cooked beans or 1 cup of pea soup, 1 apple or ½ cup oatmeal, or 1 tablespoon of avocado fruit or ½ tablespoon of canola or olive oil per day. These foods should displace foods in one’s current diet, ideally those high in saturated fat and cholesterol.

### Limitations

A major limitation of NHANES is the risk of misclassification, as diet exposure data are collected at a single baseline time point. While extreme values from the 24-h recalls were verified, dietary data were self-reported and therefore are susceptible to bias. A single (or a few) 24-h recall never reflect usual intakes for an individual. While repeated dietary recalls would allow for measurement error correction, NHANES III only includes a second 24-h recall in a small subsample (less than 8% of our study population). Given the large number of non-reporters for 3 key components of the Portfolio Diet—including nuts, plant protein from legumes, and MUFA sources—it was not advisable to apply the National Cancer Institute (NCI) method. Instead, we supplemented 24-h recall data with responses from the FFQ, which allowed us to differentiate between never-consumers and non-consumers. The combination of the 24-h recalls and the FFQ data has been done to look at ultra-processed foods [47] and the Mediterranean diet [41] and has been recommended [16].

Random measurement error is another limitation, as it affects all dietary variables assessed through 24-h recall. As noted in previous literature [48], such errors do not simply attenuate risk estimates but can bias results in any direction, particularly in multivariable models where multiple error-prone independent variables are considered jointly [49, 50]. Although state-of-the-art measurement error correction methods, such as the NCI multivariate algorithm [51], are available for modeling episodically consumed foods, it was not used in this analysis given limited subsample with repeated 24-h recalls (7% of our study population) and the extreme zero-inflation of reported intakes from the 24-h recalls for 3 of the 6 PDS categories. In the absence of such analysis, we acknowledge that our estimates may be biased due to uncorrected measurement error. Additionally, the PDS is limited given it requires the categorization of individuals for scoring, our findings should be interpreted with caution due to the potential residual bias and inefficiency [52]. Furthermore, because subgroup analyses are exploratory and the grouping of individuals into broad categories of race/ethnicity has severe limitations [53], no conclusion beyond emphasizing the necessity of future research involving underserved populations can be made.

## Conclusions

Among a representative sample of US adults, greater adherence to the Portfolio dietary pattern was inversely associated with CVD, CHD, and all-cause mortality.

## Supplementary Information

Additional file 1: Appendix 1–3; Tables S1-S10; Figures S1-S8. Appendix 1. Mortality linkage. Appendix 2. Covariate assessment. Appendix 3. Additional analyses performed beyond primary outcomes. Table S1. Scoring Criteria for the Portfolio Diet using the Portfolio Diet Score with Commonly Reported Foods Items in the 24-h recalls. Table S2. Mean intake of the Portfolio Diet components by tertiles of the Portfolio Diet Score, weighted means. Table S3. Adjusted hazard ratio of the primary outcome of CVD mortality according to tertiles of the Portfolio Diet Score by baseline CVD status. Table S4. Adjusted hazard ratio of CHD mortality according to tertiles of the Portfolio Diet Score by baseline CVD status. Table S5. Adjusted hazard ratio of stroke mortality according to tertiles of the Portfolio Diet Score by baseline CVD status. Table S6. Adjusted hazard ratio of all-cause mortality according to tertiles of the Portfolio Diet Score by baseline CVD status. Table S7. Spearman correlation coefficients between the Portfolio Diet Scoreand the Healthy Eating Index: unweighted analysis. Table S8. Adjusted hazard ratio of all mortality outcomes with BMI according to tertiles of the Portfolio Diet Score. Table S9. Adjusted associations of the individual Portfolio Diet Score components and CVD mortality through a continuous analysis using RCS fully adjusted model using linked mortality files through 2019. Table S10. Subgroup analysis for race/ethnicity by four original groupings with adjusted hazard ratio for all outcomes. Figure S1. Selection of study population. Figure S2. Directed acyclic graph of the Portfolio Diet and CVD mortality. Figure S3. Survival curves for: CVD mortality, CHD mortality,stroke mortality, and all-cause mortality. Figure S4. Adjusted differences in hazard ratio for: CVD mortality, CHD mortality, stroke mortality, and all-cause mortality. Figure S5. Subgroup analysis with adjusted hazard ratio for CVD mortality. Figure S6. Subgroup analysis with adjusted hazard ratio for CHD mortality. Figure S7. Subgroup analysis with adjusted hazard ratio for stroke mortality. Figure S8. Subgroup analysis with adjusted hazard ratio for all-cause mortality.

## Data Availability

The datasets analysed during the current study are publicly available from the CDC National Center for Health Statistics: https://wwwn.cdc.gov/nchs/nhanes/nhanes3/datafiles.aspx. The SAS analytic code used for the presented analyses is also publicly available on Github: https://github.com/MegatronKavanagh/SAS/blob/main/NHANESII_PDS_11APR2024.

## Abbreviations

ApoB: Apolipoprotein B
BMI: Body mass index
β: Beta-coefficient
CDC: Centers for Disease Control and Prevention
CIs: Confidence intervals
CHD: Coronary heart disease
CRP: C-reactive protein
CVD: Cardiovascular disease
DASH: Dietary approach to stop hypertension
DBP: Diastolic blood pressure
FFQ: Food frequency questionnaire
FNDDS: Food and Nutrient Database for Dietary Studies
G20: Group of twenty
HbA1c: Glycosylated hemoglobin or hemoglobin A1c
HDL-C: High-density lipoprotein cholesterol
HEI: Healthy eating index
HPFS: Health Professionals Follow-up Study
HR: Hazard ratio
LDL-C: Low-density lipoprotein cholesterol
Lp(a): Lipoprotein(a)
MEC: Mobile examination center
MUFA: Monounsaturated fatty acid
NCEP: National Cholesterol Education Program
NCI: National Cancer Institute
NHANES: National Health and Nutrition Examination Survey
NHS: Nurses’ Health Study
Non-HDL-C: Non-high-density lipoprotein cholesterol
PDS: Portfolio Diet Score
PSU: Primary sampling unit
RCS: Restricted cubic spline
SBP: Systolic blood pressure
SE: Standard error
SES: Social economic status
TC: Total cholesterol
TG: Triglycerides
T1: First tertile
T2: Second tertile
T3: Third tertile
US: United States
WHI: Women’s Health Initiative
