# Supplementary material for: The portfolio dietary pattern and risk of cardiovascular disease mortality during 1988–2019 in US adults: a prospective cohort study
Source: BMC Med. 2025 May 21;23:287. doi: 10.1186/s12916-025-04067-1 (PMC12093672; doi:10.1186/s12916-025-04067-1)
Supplement: Supplementary file 1 — Additional file 1: Appendix 1–3; Tables S1-S10; Figures S1-S8. Appendix 1. Mortality linkage. Appendix 2. Covariate assessment. Appendix 3. Additional analyses performed beyond primary outcomes. Table S1. Scoring Criteria for the Portfolio Diet using the Portfolio Diet Score with Commonly Reported Foods Items in the 24-h recalls. Table S2. Mean intake of the Portfolio Diet components by tertiles of the Portfolio Diet Score, weighted means. Table S3. Adjusted hazard ratio of the primary outcome of CVD mortality according to tertiles of the Portfolio Diet Score by baseline CVD status. Table S4. Adjusted hazard ratio of CHD mortality according to tertiles of the Portfolio Diet Score by baseline CVD status. Table S5. Adjusted hazard ratio of stroke mortality according to tertiles of the Portfolio Diet Score by baseline CVD status. Table S6. Adjusted hazard ratio of all-cause mortality according to tertiles of the Portfolio Diet Score by baseline CVD status. Table S7. Spearman correlation coefficients between the Portfolio Diet Scoreand the Healthy Eating Index: unweighted analysis. Table S8. Adjusted hazard ratio of all mortality outcomes with BMI according to tertiles of the Portfolio Diet Score. Table S9. Adjusted associations of the individual Portfolio Diet Score components and CVD mortality through a continuous analysis using RCS fully adjusted model using linked mortality files through 2019. Table S10. Subgroup analysis for race/ethnicity by four original groupings with adjusted hazard ratio for all outcomes. Figure S1. Selection of study population. Figure S2. Directed acyclic graph of the Portfolio Diet and CVD mortality. Figure S3. Survival curves for: CVD mortality, CHD mortality,stroke mortality, and all-cause mortality. Figure S4. Adjusted differences in hazard ratio for: CVD mortality, CHD mortality, stroke mortality, and all-cause mortality. Figure S5. Subgroup analysis with adjusted hazard ratio for CVD mortality. Figure S6. Subgroup analysis with adjuste [file 12916_2025_4067_MOESM1_ESM.docx]

**The Portfolio Dietary Pattern and Risk of Cardiovascular Disease Mortality During 1988-2019 in US Adults: A Prospective Cohort Study**

Meaghan E Kavanagh, Andreea Zurbau, Andrea J Glenn, Julianah O Oguntala, Robert G Josse, Vasanti S Malik, Laura Chiavaroli, Simin Liu, Cyril W C Kendall, David J A Jenkins, John L Sievenpiper

Table of Contents

[APPENDICES 2](#_Toc194995609)

[Appendix 1. Mortality linkage 2](#_Toc194995610)

[Appendix 2. Covariate assessment 2](#_Toc194995611)

[Appendix 3. Additional analyses performed beyond primary outcomes 3](#_Toc194995612)

[TABLES 5](#_Toc194995613)

[Table S1. Scoring Criteria for the Portfolio Diet using the Portfolio Diet Score with Commonly Reported Foods Items in the 24-hour recalls in National Health and Nutrition Examination Survey 1988-1994 5](#_Toc194995614)

[Table S2. Mean intake of the Portfolio Diet components by tertiles of the Portfolio Diet Score (PDS), weighted means (SE) in National Health and Nutrition Examination Survey 1988-1994 6](#_Toc194995615)

[Table S3. Adjusted hazard ratio of the primary outcome of CVD mortality according to tertiles of the Portfolio Diet Score by baseline CVD status: National Health and Nutrition Examination Survey Linked Mortality Files (1988-2019) 7](#_Toc194995616)

[Table S4. Adjusted hazard ratio of CHD mortality according to tertiles of the Portfolio Diet Score by baseline CVD status: National Health and Nutrition Examination Survey Linked Mortality Files (1988-2019) 8](#_Toc194995617)

[Table S5. Adjusted hazard ratio of stroke mortality according to tertiles of the Portfolio Diet Score by baseline CVD status: National Health and Nutrition Examination Survey Linked Mortality Files (1988-2019) 9](#_Toc194995618)

[Table S6. Adjusted hazard ratio of all-cause mortality according to tertiles of the Portfolio Diet Score by baseline CVD status: National Health and Nutrition Examination Survey Linked Mortality Files (1988-2019) 10](#_Toc194995619)

[Table S7. Spearman correlation coefficients between the Portfolio Diet Score (PDS) and the Healthy Eating Index (HEI): unweighted analysis 11](#_Toc194995620)

[Table S8. Adjusted hazard ratio of all mortality outcomes with body mass index according to tertiles of the Portfolio Diet Score: National Health and Nutrition Examination Survey Linked Mortality Files (1988-2019) 11](#_Toc194995621)

[Table S9. Adjusted associations of the individual Portfolio Diet Score components and CVD mortality through a continuous analysis using RCS fully adjusted model in NHANES 1988-1994 using linked mortality files through 2019 12](#_Toc194995622)

[Table S10. Subgroup analysis for race/ethnicity by four original groupings used in NHANES III with adjusted hazard ratio for all outcomes 13](#_Toc194995623)

[FIGURES 15](#_Toc194995624)

[Figure S1. Selection of study population for National Health and Nutrition Examination Survey (1988-1994). 15](#_Toc194995625)

[Figure S2. Directed acyclic graph of the Portfolio Diet and CVD mortality 16](#_Toc194995626)

[Figure S3. Adjusted survival curves for: (a) CVD mortality, (b) CHD mortality, (c) stroke mortality, and (d) all-cause mortality 17](#_Toc194995627)

[Figure S4. Adjusted differences in hazard ratio for: (a) CVD mortality, (b) CHD mortality, (c) stroke mortality, and (d) all-cause mortality 18](#_Toc194995628)

[Figure S5. Subgroup analysis with adjusted hazard ratio for CVD mortality 19](#_Toc194995629)

[Figure S6. Subgroup analysis with adjusted hazard ratio for CHD mortality 21](#_Toc194995630)

[Figure S7. Subgroup analysis with adjusted hazard ratio for stroke mortality 22](#_Toc194995631)

[Figure S8. Subgroup analysis with adjusted hazard ratio for all-cause mortality 23](#_Toc194995632)

# **APPENDICES**

# **Appendix 1.** Mortality linkage

***NHANES III (1988-1994) Linked Mortality File (1988-2019)***

To determine mortality status for linkage-eligible participants in NHANES III, participants were matched to the National Death Index from October 18^th^, 1988, through December 31^st^, 2019, through deterministic and probabilistic approaches. For cardiovascular disease (CVD), follow-up continued until death attributable to CVD, with censoring at the time of death for those who died from causes other than CVD. Those not matched with a death record were considered alive for the entire follow-up period. A detailed description of this methodology has been published (24). The National Center for Health Statistics identified underlying causes of death for participants based on the *International Classification of Diseases, Tenth Revision codes* (ICD-10), with CVD identified as Disease of heart (ICD codes: I00-I09, I11, I13, I20-I51) and Cerebrovascular disease (ICD codes I60-I69). We defined CHD mortality as deaths identified as Disease of heart (ICD codes: I00-I09, I11, I13, I20-I51) and stroke mortality as deaths identified as Cerebrovascular disease (ICD codes I60-I69). Events that occurred within the first year were censored.

# **Appendix 2.** Covariate assessment

***Covariate assessments***

Race and ethnicity was classified based on the NHANES survey design into four categories: non-Hispanic White adults, non-Hispanic Black adults, Mexican American adults, and other which included all other Hispanics regardless of race (such as Other Latin American/Spanish ancestry or national origin) and also all other non-Hispanic adults from racial groups other than White or Black (i.e., American Indian or Alaskan Native; Native Hawaiian or Pacific Islander; multiple races or ethnicities; or unknown). PIR was computed as a ratio of two components, both the observed family income and the poverty threshold values at the time of interview.

Model 1 included the following covariates: age (continuous), sex (male, female), and reported race/ethnicity (non-Hispanic White; non-Hispanic Black; Mexican American; other). Sex, age, and race/ethnicity are key variables used in survey weighting of NHANES. Commonly used for the minimally adjusted model as adjusting for them ensures that analyses correctly represent the U.S. population. Model 2 included Model 1 and additional covariates identified from a direct acyclic graph constructed from literature and expert knowledge: educational attainment (less than high school [<12 years], high school graduate [12 years], and more than high school [>12 years]), smoking status (never, former, current), Poverty Income Ratio (PIR) (low [<1.3], middle [1.3 to 3.5], high [>3.5]), marital status (married or living with partner, divorced/widowed/separated, never married), family history of CVD (yes/no), physical activity (0, <5, or ≥5 times/week of moderate-intensity to vigorous activities), alcohol consumption (0, <3, or ≥3 drinks/week), awareness of hypertension (yes/no), awareness of a type 2 diabetes mellitus (T2D) diagnosis (yes/no). Model 3 included Model 2 and additional dietary covariates that may confound the relationship between the Portfolio Diet Score (PDS) and CVD outcomes: energy intake (continuous), and sodium (continuous). Other dietary variables such as fibre were considered but were not included given their overlap with the Portfolio Diet. Using multiple models allows for a stepwise adjustment approach, progressively accounting for potential confounders and examining how the association between the Portfolio Diet Score (PDS) and CVD outcomes changes with additional adjustments and also ensures comparability with prior studies.

Those with self-reported type 2 diabetes were included in the sample as these were considered on the causal pathway, with a subgroup analysis planned. As dietary intake often changes after diagnosis of a clinical condition, we adjusted for any clinical conditions participants were aware of (self-reported physician diagnosed T2D and hypertension), but not those which could be inferred based on health measures collected in the MEC visit. Only those who responded “yes” to ever having their blood cholesterol checked were asked if they had high cholesterol. Therefore, there was a high number of missing responses for self-reported hypercholesterolemia (7,104) and cholesterol-lowering medication (14,190), it was decided to plan for sensitivity analyses for self-reported hypercholesterolemia. Body mass index (BMI) was not adjusted in the primary analysis, to allow for exploration of its possible mediating role in the association between the Portfolio Diet Score (PDS) and mortality outcomes.

# **Appendix 3.** Additional analyses performed beyond primary outcomes

Sensitivity analyses were conducted where those without CVD at baseline (primary prevention) and those with CVD at baseline (secondary prevention) were assessed separately with concerns of reverse-causality. We also assessed the mediation of BMI by the magnitude of attenuation in the associated HR after the addition of BMI (continuous variable) to the fully adjusted model.

We performed a stratified analysis to look at pre-determined exploratory subgroups for adjusted HR of mortality outcomes by sex, age (<60 years vs. ≥60 years), race/ethnicity (White [includes reported non-Hispanic White], non-White [includes reported non-Hispanic Black, Mexican American, and other]), educational attainment (<12 vs. ≥12 years), PIR (low, mid, and high), physical activity (high [≥5 times/wk of moderate-intensity to vigorous activities] vs. low [<5 times/wk]), smoking status (current, former, and never), T2D (no vs. yes), BMI (<30 vs. ≥30 kg/m^2^), hypertension (no vs. yes), and hypercholesteremia (no vs. yes), comparing the first to the third tertile of PDS. Race/ethnicity groups were consolidated owing to small number of events. Interaction terms were included in the Cox proposal hazards model using the PROC SURVEYPHREG procedure to account for complex sample design and significance of interactions terms were assessed by Wald chi-squared test while adjusting for known CVD covariates with a *P*<0.05 considered significant. However, to algin with recommendations from the American Statistical Association who discourage the dichotomization of p-values (e.g. “<0.05” or “nonsignificant”) associated estimates and uncertainties were interpreted in context rather than relying on a strict *P*-value threshold (27). We used the DOMAIN statement within the PROC SURVEYPHREG procedure to obtain HRs and CIs within each subgroup allowing variance estimates to appropriately reflect the complex survey design.

Linear regression analyses were performed in a cross-sectional analysis between the PDS and biomarkers of cardiometabolic health (lipids and inflammation). Prior to analysis, univariate statistics of all dependent variables were assessed and those which were extremely non-normal were log-transformed to an approximately normal distribution (CRP, triglycerides, and blood pressure). We used Spearman correlation coefficients to assess the overlap of the PDS with the Healthy Eating Index (HEI), to assess overlap . We also assessed the six individual components of the PDS (nuts and seeds, plant protein, viscous fiber, phytosterols, plant MUFA food sources, and high saturated fat and cholesterol foods) using RCS with, and without, adjustments for the five other components.

# **TABLES**

# **Table S1.** Scoring Criteria for the Portfolio Diet using the Portfolio Diet Score with Commonly Reported Foods Items in the 24-hour recalls in National Health and Nutrition Examination Survey 1988-1994

| **PDS foods** | | **Food items from 24-hour dietary recalls, standard serving size in grams** |
| --- | --- | --- |
|  | Nuts and seeds, servings | Peanut butter, 32g; peanuts, 28g; sunflower seeds, 30g; cashew nuts, 28g; pecans, 28g; pumpkin and/or squash seeds, 10g; mixed nuts, 30g; |
|  | Plant Protein, servings | Refried beans, 130g; peas, green, 80g; red beans and rice, 170g; lima beans, 92g; pea soup, 250g; baked beans with tomato sauce, 130g; |
|  | Viscous Fiber foods, servings | Apple, raw, 200g, applesauce, 123g; orange, raw, 154g; blueberries, 75g; oatmeal, 120g; bread, oatbran, 28g; Cracklin’ Oat Bran, 73g; okra, 83g; eggplant, 50g; |
|  | Plant Sterols, mg | Estimated from all foods from available databases* |
|  | MUFA foods, servings | Avocado, 30g; guacamole, 60g; Italian dressing, 29g; olive oil, 14g; safflower oil, 14g; sunflower oil, 14g; |
|  | High saturated fat and cholesterol foods, servings | Cow’s milk whole, 244g; cheese processed, 21g; egg, whole, 50g; pork bacon, 16g; butter, 14g; ice cream, 135g; pork sausage, 85g; ground beef, 80g; beef steak, 134g; chicken, with skin, 105g; |
| Abbreviations: MUFA, monounsaturated fatty acids; PDS, Portfolio Diet Score; T1 first tertile; T2, second tertile; T3, third tertile; SE, Standard Error.  *Finnish Food Composition Database, the European Prospective Cohort into Cancer (EPIC) Netherlands database, and the United States Department of Agriculture (USDA). | | |

|  |
| --- |

# **Table S2.** Mean intake of the Portfolio Diet components by tertiles of the Portfolio Diet Score (PDS), weighted means (SE) in National Health and Nutrition Examination Survey 1988-1994

| **Characteristic** | | **T1 (lowest)** | **T2 (mid)** | **T3 (highest)** | ***P* Value*** |
| --- | --- | --- | --- | --- | --- |
| **No.** | | 4,832 | 4,251 | 5,752 |  |
| **Dietary Score, mean (SE)** | | 14.0 (0.04) | 18.0 (0.02) | 22.1 (0.04) | <0.001 |
| **Portfolio Diet Foods** | |  |  |  |  |
|  | Nuts and seeds, mean (SE), servings/day | 0.12 (0.01) | 0.29 (0.01) | 0.61 (0.03) | <0.001 |
|  | Plant Protein, mean (SE), servings/day | 0.20 (0.02) | 0.45 (0.02) | 1.03 (0.15) | <0.001 |
|  | Viscous Fiber foods, mean (SE), servings/day | 0.22 (0.01) | 0.50 (0.02) | 1.01 (0.03) | <0.001 |
|  | Plant Sterols, mean (SE), mg/day | 214.67 (3.08) | 263.31 (4.70) | 339.19 (5.09) | <0.001 |
|  | MUFA foods, mean (SE), servings/day | 0.17 (0.01) | 0.30 (0.02) | 0.49 (0.02) | <0.001 |
|  | High saturated fat foods, mean (SE), servings/day | 4.37 (0.07) | 3.71 (0.08) | 2.93 (0.09) | <0.001 |
| Abbreviations: MUFA, monounsaturated fatty acids; PDS, Portfolio Diet Score; T1, first tertile; T2, second tertile; T3, third tertile; SE, Standard Error.  * *P* values are presented for difference across categories of Portfolio Diet. All tests were 2-tailed. | | | | | |

# **Table S3.** Adjusted hazard ratio of the primary outcome of CVD mortality according to tertiles of the Portfolio Diet Score by baseline CVD status: National Health and Nutrition Examination Survey Linked Mortality Files (1988-2019)

|  | | | | | | |
| --- | --- | --- | --- | --- | --- | --- |
| **Characteristic** | | **T1 (low)** | **T2 (medium)** | **T3 (high)** | ***P* value**  **_for trend_** | **per 8-points, HR (95% CI)** |
| **No CVD at baseline** | | | | | | |
|  | Cases | 550 | 504 | 697 |  |  |
|  | Participants | 4,422 | 3,896 | 5,268 |  |  |
|  | Person years | 101,697 | 90,069 | 120,061 |  |  |
| HR (95% CI) | |  |  |  |  |  |
|  | Adjusted only for age, sex, race/ethnicity | 1 [Ref] | 0.83 (0.71, 0.98) | 0.72 (0.61, 0.84) | <0.001 | 0.74 (0.64, 0.85) |
|  | Model 2 | 1 [Ref] | 0.88 (0.73, 1.04) | 0.83 (0.72, 0.96) | 0.015 | 0.85 (0.74, 0.98) |
|  | Model 3 | 1 [Ref] | 0.88 (0.73, 1.04) | 0.84 (0.72, 0.98) | 0.027 | 0.86 (0.75, 0.99) |
| **CVD at baseline** | | | | | | |
|  | Cases | 181 | 148 | 220 |  |  |
|  | Participants | 410 | 355 | 484 |  |  |
|  | Person years | 4,700 | 4,260 | 5,757 |  |  |
| HR (95% CI) | |  |  |  |  |  |
|  | Adjusted only for age, sex, race/ethnicity | 1 [Ref] | 0.89 (0.67, 1.18) | 0.89 (0.66, 1.20) | 0.46 | 0.90 (0.70, 1.18) |
|  | Model 2 | 1 [Ref] | 0.81 (0.62, 1.07) | 0.85 (0.63, 1.14) | 0.32 | 0.90 (0.70, 1.15) |
|  | Model 3 | 1 [Ref] | 0.87 (0.63, 1.15) | 0.93 (0.69, 1.24) | 0.68 | 0.96 (0.75, 1.23) |
| **P value for interaction on CVD status** | | 0.98 |  |  |  |  |
| Abbreviations: CI, confidence intervals; CVD, cardiovascular disease; HR, hazard ratio; T1, first tertile; T2, second tertile; T3, third tertile; SE, Standard Error.  Model 2: age, sex, and race/ethnicity, educational attainment, smoking status, Poverty Income Ratio, marital status, physical activity, alcohol consumption, family history of CVD, self-reported cancer (other than skin), self-reported T2D, and self-reported hypertension.  Model 3: age, sex, and race/ethnicity, educational attainment, smoking status, Poverty Income Ratio, marital status, physical activity, alcohol consumption, family history of CVD, self-reported cancer (other than skin), self-reported type-2 diabetes, self-reported hypertension, energy intake, and sodium. | | | | | | |

# **Table S4.** Adjusted hazard ratio of CHD mortality according to tertiles of the Portfolio Diet Score by baseline CVD status: National Health and Nutrition Examination Survey Linked Mortality Files (1988-2019)

|  | | | | | | | |
| --- | --- | --- | --- | --- | --- | --- | --- |
| **Characteristic** | | **T1 (low)** | | **T2 (medium)** | **T3 (high)** | ***P* value**  **_for trend_** | **per 8-points, HR (95% CI)** |
| **No CVD at baseline** | | | | | | | |
|  | Cases | 449 | 419 | | 548 |  |  |
|  | Participants | 4,422 | 3,896 | | 5,268 |  |  |
|  | Person years | 101,697 | 90,069 | | 120,061 |  |  |
| HR (95% CI) | |  |  | |  |  |  |
|  | Adjusted only for age, sex, race/ethnicity | 1 [Ref] | 0.81 (0.68, 0.96) | | 0.69 (0.58, 0.82) | <0.001 | 0.69 (0.59, 0.81) |
|  | Model 2 | 1 [Ref] | 0.86 (0.72, 1.02) | | 0.81 (0.70, 0.94) | 0.010 | 0.82 (0.71, 0.94) |
|  | Model 3 | 1 [Ref] | 0.87 (0.71, 1.07) | | 0.82 (0.70, 0.95) | 0.015 | 0.82 (0.72, 0.95) |
| **CVD at baseline** | | | | | | | |
|  | Cases | 153 | 124 | | 194 |  |  |
|  | Participants | 410 | 355 | | 484 |  |  |
|  | Person years | 4,700 | 4,260 | | 5,757 |  |  |
| HR (95% CI) | |  |  | |  |  |  |
|  | Adjusted only for age, sex, race/ethnicity | 1 [Ref] | 0.90 (0.66, 1.21) | | 0.89 (0.67, 1.20) | 0.46 | 0.93 (0.72, 1.21) |
|  | Model 2 | 1 [Ref] | 0.80 (0.59, 1.09) | | 0.84 (0.63, 1.12) | 0.30 | 0.92 (0.72, 1.18) |
|  | Model 3 | 1 [Ref] | 0.86 (0.63, 1.17) | | 0.93 (0.70, 1.23) | 0.70 | 0.99 (0.77, 1.28) |
|  | **P value for interaction on CVD status** | 0.33 |  | |  |  |  |
| Abbreviations: CI, confidence intervals; CHD, coronary heart disease; CVD, cardiovascular disease; HR, hazard ratio; T1 first tertile; T2, second tertile; T3, third tertile; SE, Standard Error.  Model 2: age, sex, and race/ethnicity, educational attainment, smoking status, Poverty Income Ratio, marital status, physical activity, alcohol consumption, family history of CVD, self-reported cancer (other than skin), self-reported T2D, and self-reported hypertension.  Model 3: age, sex, and race/ethnicity, educational attainment, smoking status, Poverty Income Ratio, marital status, physical activity, alcohol consumption, family history of CVD, self-reported cancer (other than skin), self-reported type-2 diabetes, self-reported hypertension, energy intake, and sodium. | | | | | | | |

# **Table S5.** Adjusted hazard ratio of stroke mortality according to tertiles of the Portfolio Diet Score by baseline CVD status: National Health and Nutrition Examination Survey Linked Mortality Files (1988-2019)

|  | | | | | | |
| --- | --- | --- | --- | --- | --- | --- |
| **Characteristic** | | **T1 (low)** | **T2 (medium)** | **T3 (high)** | ***P* value**  **_for trend_** | **per 8-points, HR (95% CI)** |
| **No CVD at baseline** | | | | | | |
|  | Cases | 101 | 85 | 149 |  |  |
|  | Participants | 4,422 | 3,896 | 5,268 |  |  |
|  | Person years | 101,697 | 90,069 | 120,061 |  |  |
| HR (95% CI) | |  |  |  |  |  |
|  | Adjusted only for age, sex, race/ethnicity | 1 [Ref] | 0.95 (0.60, 1.50) | 0.90 (0.60, 1.34) | 0.60 | 1.00 (0.74, 1.36) |
|  | Model 2 | 1 [Ref] | 0.95 (0.58, 1.45) | 0.91 (0.58, 1.45) | 0.70 | 1.04 (0.73, 1.47) |
|  | Model 3 | 1 [Ref] | 0.95 (0.57, 1.58) | 0.94 (0.59, 1.50) | 0.80 | 1.07 (0.74, 1.53) |
| **CVD at baseline** | | | | | | |
|  | Cases | 28 | 24 | 26 |  |  |
|  | Participants | 410 | 355 | 484 |  |  |
|  | Person years | 4,700 | 4,260 | 5,757 |  |  |
| HR (95% CI) | |  |  |  |  |  |
|  | Adjusted only for age, sex, race/ethnicity | 1 [Ref] | 0.85 (0.35, 2.03) | 0.86 (0.39, 1.92) | 0.72 | 0.72 (0.42, 1.23) |
|  | Model 2 | 1 [Ref] | 1.37 (0.56, 3.32) | 0.93 (0.37, 2.34) | 0.69 | 0.73 (0.41, 1.33) |
|  | Model 3 | 1 [Ref] | 0.99 (0.44, 2.20) | 0.93 (0.42, 2.06) | 0.85 | 0.77 (0.43, 1.39) |
| **P value for interaction on CVD status** | | 0.67 |  |  |  |  |
| Abbreviations: CI, confidence intervals; CVD, cardiovascular disease; HR, hazard ratio; T1 first tertile; T2, second tertile; T3, third tertile; SE, Standard Error.  Model 2: age, sex, and race/ethnicity, educational attainment, smoking status, Poverty Income Ratio, marital status, physical activity, alcohol consumption, family history of CVD, self-reported cancer (other than skin), self-reported T2D, and self-reported hypertension.  Model 3: age, sex, and race/ethnicity, educational attainment, smoking status, Poverty Income Ratio, marital status, physical activity, alcohol consumption, family history of CVD, self-reported cancer (other than skin), self-reported type-2 diabetes, self-reported hypertension, energy intake, and sodium. | | | | | | |

# **Table S6.** Adjusted hazard ratio of all-cause mortality according to tertiles of the Portfolio Diet Score by baseline CVD status: National Health and Nutrition Examination Survey Linked Mortality Files (1988-2019)

| **Characteristic** | | **T1 (low)** | **T2 (medium)** | **T3 (high)** | ***P* value**  **_for trend_** | | **per 8-points, HR (95% CI)** |
| --- | --- | --- | --- | --- | --- | --- | --- |
| **No CVD at baseline** | | | | | | | |
|  | Cases | 1,676 | 1,461 | 2,065 | |  |  |
|  | Participants | 4,422 | 3,896 | 5,268 | |  |  |
|  | Person years | 101,697 | 90,069 | 120,061 | |  |  |
| HR (95% CI) | |  |  |  | |  |  |
|  | Adjusted only for age, sex, race/ethnicity | 1 [Ref] | 0.85 (0.75, 0.96) | 0.74 (0.66, 0.83) | | <.0001 | 0.75 (0.68, 0.82) |
|  | Model 2 | 1 [Ref] | 0.89 (0.80, 1.00) | 0.86 (0.77, 0.96) | | 0.009 | 0.87 (0.79, 0.95) |
|  | Model 3 | 1 [Ref] | 0.88 (0.80, 0.99) | 0.86 (0.77, 0.96) | | 0.009 | 0.87 (0.80, 0.95) |
| **CVD at baseline** | |  |  |  | |  |  |
|  | Cases | 345 | 292 | 399 | |  |  |
|  | Participants | 410 | 355 | 484 | |  |  |
|  | Person years | 4,700 | 4,260 | 5,757 | |  |  |
| HR (95% CI) | |  |  |  | |  |  |
|  | Adjusted only for age, sex, race/ethnicity | 1 [Ref] | 0.92 (0.76, 1.17) | 0.89 (0.66, 1.19) | | 0.42 | 0.90 (0.73, 1.11) |
|  | Model 2 | 1 [Ref] | 0.88 (0.68, 1.12) | 0.88 (0.64, 1.20) | | 0.40 | 0.92 (0.75, 1.14) |
|  | Model 3 | 1 [Ref] | 0.90 (0.71, 1.14) | 0.92 (0.70, 1.22) | | 0.61 | 0.97 (0.80, 1.17) |
| **P value for interaction on CVD status** | | 0.68 |  |  | |  |  |
| Abbreviations: CI, confidence intervals; CVD, cardiovascular disease; HR, hazard ratio; NHANES, National Health and Nutrition Examination Survey; T1 first tertile; T2, second tertile; T3, third tertile; SE, Standard Error.  Model 2: age, sex, and race/ethnicity, educational attainment, smoking status, Poverty Income Ratio, marital status, physical activity, alcohol consumption, family history of CVD, self-reported cancer (other than skin), self-reported T2D, and self-reported hypertension.  Model 3: age, sex, and race/ethnicity, educational attainment, smoking status, Poverty Income Ratio, marital status, physical activity, alcohol consumption, family history of CVD, self-reported cancer (other than skin), self-reported type-2 diabetes, self-reported hypertension, energy intake, and sodium. | | | | | | | |

# **Table S7.** Spearman correlation coefficients between the Portfolio Diet Score (PDS) and the Healthy Eating Index (HEI): unweighted analysis

| **Dietary Pattern** | **Mean (SD)** | **Min** | **Max** | **r*** |
| --- | --- | --- | --- | --- |
| HEI | 63.0 (13.3) | 10 | 99.2 |  |
| PDS | 18.4 (3.9) | 6 | 30 | 0.35 |

Abbreviations: HEI, healthy eating index; SD, standard deviation; PDS, Portfolio Diet Score.

Spearman correlation analysis was unweighted.

# **Table S8.** Adjusted hazard ratio of all mortality outcomes with body mass index according to tertiles of the Portfolio Diet Score: National Health and Nutrition Examination Survey Linked Mortality Files (1988-2019)

| **Outcome** | | **T1 (low)** | **T2 (medium)** | **T3 (high)** | ***P* value**  **_for trend_** | **HR (95% CI)**  **_for 8-points_**  **_(33-percentile increments)_** |
| --- | --- | --- | --- | --- | --- | --- |
| **All participants** | | | | | | |
| **HR (95% CI)** | |  | **HR (95% CI)** | **HR (95% CI)** |  |  |
| CVD mortality | Model 3 + BMI | 1 [Ref] | 0.86 (0.75, 0.99) | 0.86 (0.75, 0.99) | 0.049 | 0.88 (0.79, 1.02*)* |
| CHD mortality | Model 3 + BMI | 1 [Ref] | 0.84 (0.71, 0.98) | 0.84 (0.74, 0.96) | 0.020 | 0.87 (0.78, 0.98) |
| Stroke mortality | Model 3 + BMI | 1 [Ref] | 0.94 (0.60, 1.50) | 0.96 (0.62, 1.48) | 0.860 | 1.04 (0.75, 1.44) |
| All-cause mortality | Model 3 + BMI | 1 [Ref] | 0.88 (0.80, 0.96) | 0.87 (0.79, 0.97) | 0.015 | 0.89 (0.82, 0.97) |

Abbreviations: BMI, body mass index; CI, confidence intervals; CHD, coronary heart disease; CVD, cardiovascular disease; HR, hazard ratio; NHANES, National Health and Nutrition Examination Survey; T1, first tertile; T2, second tertile; T3, third tertile; SE, Standard Error.

Model 3: Fully adjusted model including age, sex, and race/ethnicity, educational attainment, smoking status, Poverty Income Ratio, marital status, physical activity, alcohol consumption, family history of CVD, self-reported history of cancer (other than skin), self-reported type-2 diabetes, self-reported hypertension, energy intake, and sodium.

BMI was adjusted for as a continuous variable (kg/m^2^)

# **Table S9.** Adjusted associations of the individual Portfolio Diet Score components and CVD mortality through a continuous analysis using RCS fully adjusted model in NHANES 1988-1994 using linked mortality files through 2019

| **HR (95% CI)** | | **T1** | **T2** | **T3** |
| --- | --- | --- | --- | --- |
| **Nuts and Seeds, mean (SE), servings/day** | | **0.12 (0.01)** | **0.29 (0.01)** | **0.61 (0.03)** |
|  | Model 1 | 1 [Ref] | 1.00 (0.91, 1.11) | 0.99 (0.88, 1.12) |
|  | Model 2 | 1 [Ref] | 1.01 (0.92, 1.12) | 1.00 (0.89, 1.12) |
| **Plant Protein, mean (SE), servings/day** | | **0.20 (0.02)** | **0.45 (0.02)** | **1.03 (0.15)** |
|  | Model 1 | 1 [Ref] | 0.91 (0.82, 1.02) | 0.86 (0.72, 1.03) |
|  | Model 2 | 1 [Ref] | 0.92 (0.82, 1.02) | 0.87 (0.73, 1.04) |
| **Viscous Fiber foods, mean (SE), servings/day** | | **0.22 (0.01)** | **0.50 (0.02)** | **1.01 (0.03)** |
|  | Model 1 | 1 [Ref] | 0.94 (0.82, 1.07) | 0.88 (0.73, 1.08) |
|  | Model 2 | 1 [Ref] | 0.94 (0.82, 1.07) | 0.88 (0.73, 1.08) |
| **Plant Sterols, mean (SE), mg/day** | | **214.67 (3.08)** | **263.31 (4.70)** | **339.19 (5.09)** |
|  | Model 1 | 1 [Ref] | 0.90 (0.81, 1.01) | 0.87 (0.73, 1.04) |
|  | Model 2 | 1 [Ref] | 0.92 (0.82, 1.02) | 0.90 (0.76, 1.07) |
| **MUFA foods, mean (SE), servings/day** | | **0.17 (0.01)** | **0.30 (0.02)** | **0.49 (0.02)** |
|  | Model 1 | 1 [Ref] | 1.02 (0.93 1.12) | 1.03 (0.91,1.17) |
|  | Model 2 | 1 [Ref] | 1.03 (0.94 1.13) | 1.04 (0.92,1.18) |
| **High saturated fat and cholesterol foods, mean (SE), servings/day** | | **4.37 (0.07)** | **3.71 (0.08)** | **2.93 (0.09)** |
|  | Model 1 | 1 [Ref] | 0.98 (0.96, 1.00) | 0.96 (0.88, 1.05) |
|  | Model 2 | 1 [Ref] | 0.98 (0.96, 1.01) | 0.97 (0.89, 1.06) |
| Abbreviations: CI, confidence intervals; CVD, cardiovascular disease; HR, hazard ratio; MUFA, monounsaturated fatty acids; T1, first tertile; T2, second tertile; T3, third tertile; SE, Standard Error.  Adjusted associations of the individual Portfolio Diet components and CVD mortality were coded using restricted cubic spline (RCS) function. In this continuous analysis the reference value for each component used was the mean servings of that component for T1 (i.e. 0.12 servings for nuts).  **Model 1:** Fully adjusted model including age, sex, and race/ethnicity, educational attainment, smoking status, Poverty Income Ratio, marital status, physical activity, alcohol consumption, family history of CVD, self-reported history of cancer (other than skin), self-reported type-2 diabetes, self-reported hypertension, energy intake, and sodium.  **Model 2:** Fully adjusted model + five other Portfolio Diet components. | | | | |

# **Table S10.** Subgroup analysis for race/ethnicity by four original groupings used in NHANES III with adjusted hazard ratio for all outcomes

| **Race/ethnicity** | **Participants No.** | **Events  No.** | **Adjusted HR (95% CI)** | **P_interaction_ by subgroup** |
| --- | --- | --- | --- | --- |
| **CVD mortality** |  |  |  | 0.001 |
| White | 4,440 | 936 | 0.92 [ 0.77, 1.11] |  |
| Black | 3,038 | 395 | 0.78 [ 0.60, 1.01] |  |
| Mexican American | 2,701 | 276 | 0.74 [ 0.51, 1.08] |  |
| Other^*^ | 405 | 41 | 0.22 [ 0.10, 0.48] |  |
| **CHD mortality** |  |  |  |  |
| White | 4,440 | 779 | 0.91 [ 0.77, 1.07] | 0.001 |
| Black | 3,038 | 317 | 0.75 [ 0.57, 0.97] |  |
| Mexican American | 2,701 | 209 | 0.86 [ 0.54, 1.36] |  |
| Other^*^ | 405 | 39 | 0.19 [ 0.08, 0.43] |  |
| **Stroke mortality** |  |  |  | 0.55 |
| White | 4,440 | 157 | 0.98 [ 0.58, 1.67] |  |
| Black | 3,038 | 78 | 0.93 [ 0.57, 1.51] |  |
| Mexican American | 2,701 | 67 | 0.47 [ 0.27, 0.81] |  |
| Other^*^ | 405 | 2 | 0.06 [ 0.00, 81.42] |  |
| **All cause mortality** |  |  |  | 0.32 |
| White | 4,440 | 2,375 | 0.90 [ 0.79, 1.03] |  |
| Black | 3,038 | 1,165 | 0.79 [ 0.70, 0.90] |  |
| Mexican American | 2,701 | 835 | 0.88 [ 0.74, 1.05] |  |
| Other^*^ | 405 | 110 | 0.55 [ 0.28, 1.08] |  |

Comparing Tertile 3 (highest) with Tertile 1 (lowest) of Portfolio Diet Score by Selected Characteristics Among US Adults 20 Years or Older: National Health and Nutrition Examination Survey (NHANES) Linked Mortality Files, 1988-2019.

Abbreviations: CI, confidence intervals; HR, Hazard Ratio.

*P* value is for interaction across subgroups for all tertiles.

^*^The other race/ethnicity includes all other Hispanic adults regardless of race (such as Other Latin American/Spanish ancestry or national origin) and also all other non-Hispanics from racial groups other than White or Black (i.e., American Indian or Alaskan Native; Native Hawaiian, Asia, or Pacific Islander; multiple races or ethnicities; or unknown).

# **FIGURES**

Full NHANES III sample

n= 33,994

<20 years old or pregnant

n= 15,457

Ineligible for mortality
and diet analysis

Adults (≥20 yrs)

n= 18,537

n=2,850

n=15,687

BMI < 18.5 kg/m^2^

n= 369

n=15,318

Missing covariates

n=483

Adults eligible for mortality and diet analysis

**n=** **14,835**

# **Figure S1.** Selection of study population for National Health and Nutrition Examination Survey (1988-1994).

The NHANES had a total of 33,994 participants. We excluded participants who were under the age of 20 years (n=15,169), those who were pregnant at baseline (n=288), who were ineligible for mortality (n=19) or diet analysis (n=2,831), or those who had a BMI under 18.5 kg/m^2^ (n=369). Finally, those with missing covariates for the multivariate analysis were also excluded (n=483). Final sample was 14,835.

**
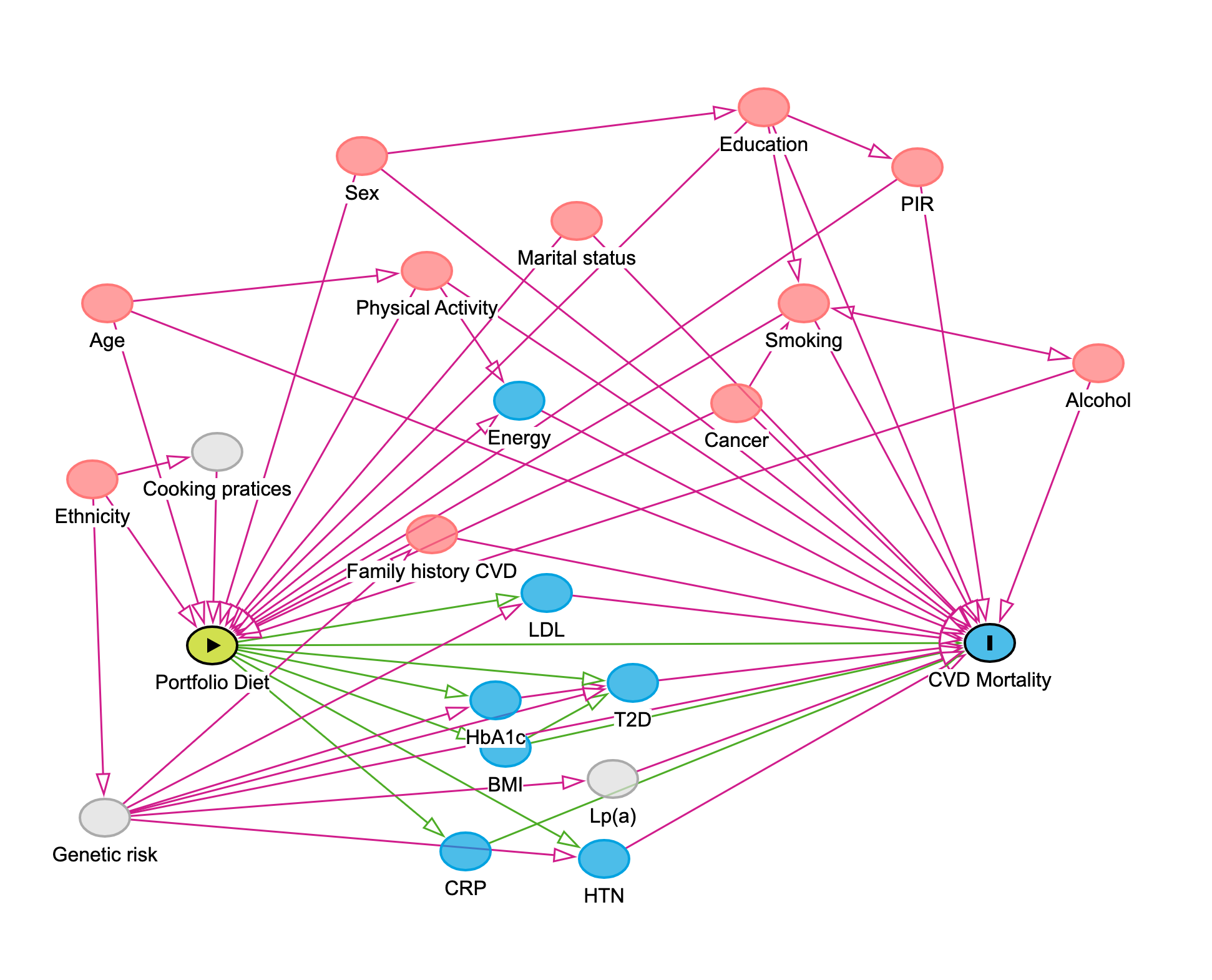
**

**Figure S2.** Directed acyclic graph of the Portfolio Diet and CVD mortality

The directed acyclic graph of the Portfolio Diet and CVD mortality was developed based on literature and expert knowledge. Nodes represent variables and arrows represent associations between variables with direct and indirect relationships. Created using the DAGitty web application (33).

Abbreviations: BMI, Body mass index; CRP, C-reactive protein; CVD; cardiovascular disease; HTN, hypertension; HDL-C, HbA1c, hemoglobin A1C; high density lipoprotein cholesterol; LDL-C, low-density lipoprotein cholesterol; Lp(a), lipoprotein (a); PIR, Poverty Income Ratio; T2D, Type-2 diabetes; T1, first tertile; T2, second tertile; T3, third tertile; SE, Standard Error.


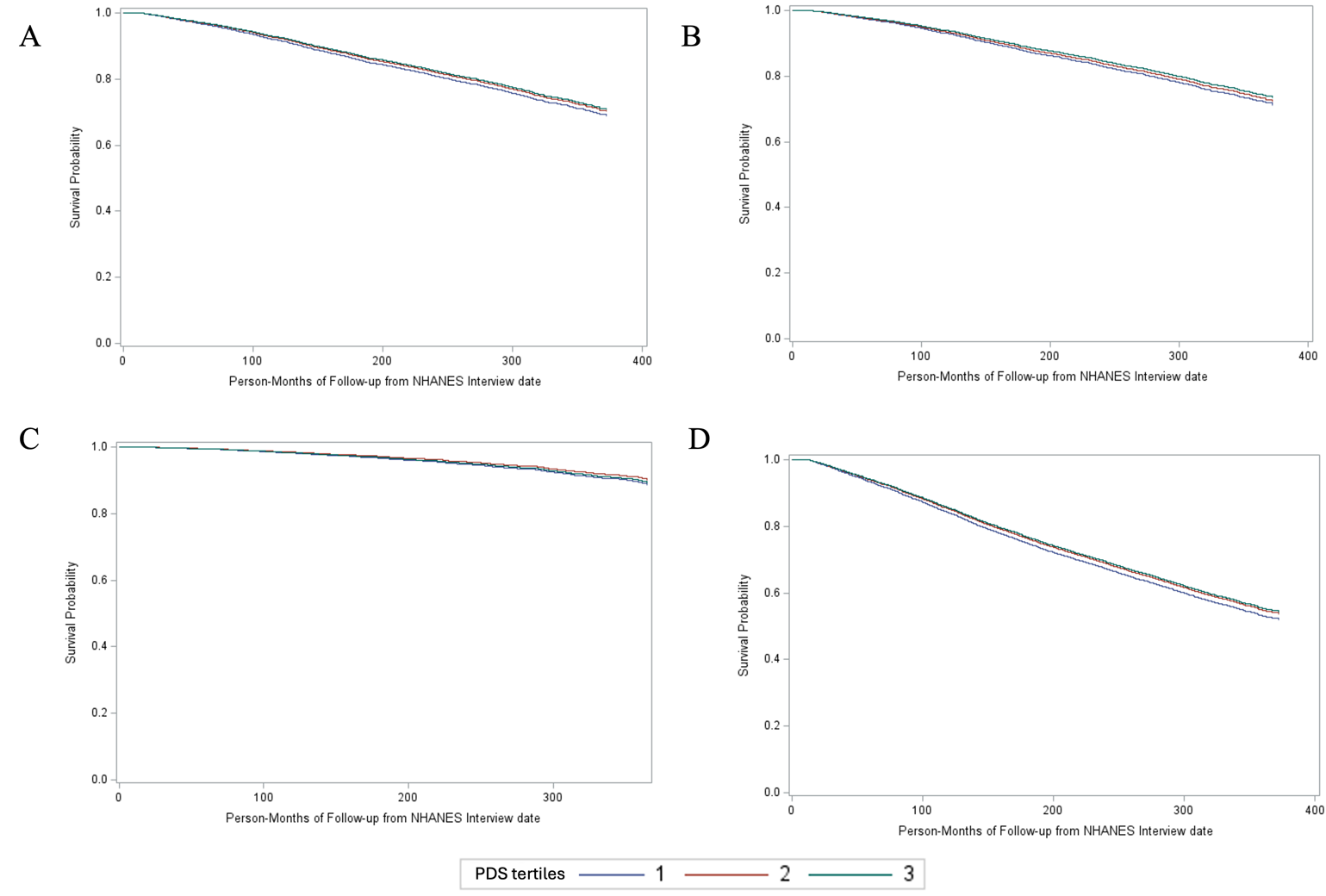


# **Figure S3.** Adjusted survival curves for: (a) CVD mortality, (b) CHD mortality, (c) stroke mortality, and (d) all-cause mortality

Abbreviations: CVD, cardiovascular disease; CHD, coronary heart disease

Survival is show over duration of follow up from the initial interview until censoring or death. Participants were matched to the National Death Index from October 18^th^, 1988, through December 31^st^, 2019. For each outcome follow-up continued until death attributable to the outcome of interest, with censoring at the time of death for those who died from causes other than outcome specified. Those not matched with a death record were considered alive for the entire follow-up period. Adjusted survival curves were derived using the BASELINE statement with the DIRADJ (direct adjustment) option, which computed model-based survival estimates standardized to the covariate distribution of the study population. Survival curves are presented for each Portfolio Diet tertile using the PLOTS(OVERLAY)=SURVIVAL option to compare risk-adjusted survival across groups.

Estimates were adjusted by age, sex, and race/ethnicity, educational attainment, smoking status, PIR, marital status, physical activity, alcohol consumption, family history of CVD, self-reported cancer (other than skin), self-reported type-2 diabetes, self-reported hypertension, energy intake, and sodium.

CVD mortality defined as deaths identified as Disease of heart (ICD codes: I00-I09, I11, I13, I20-I51) and Cerebrovascular disease (ICD codes I60-I69). CHD mortality defined as deaths identified as Disease of heart (ICD codes: I00-I09, I11, I13, I20-I51). Stroke mortality defined as deaths identified as Cerebrovascular disease (ICD codes I60-I69)


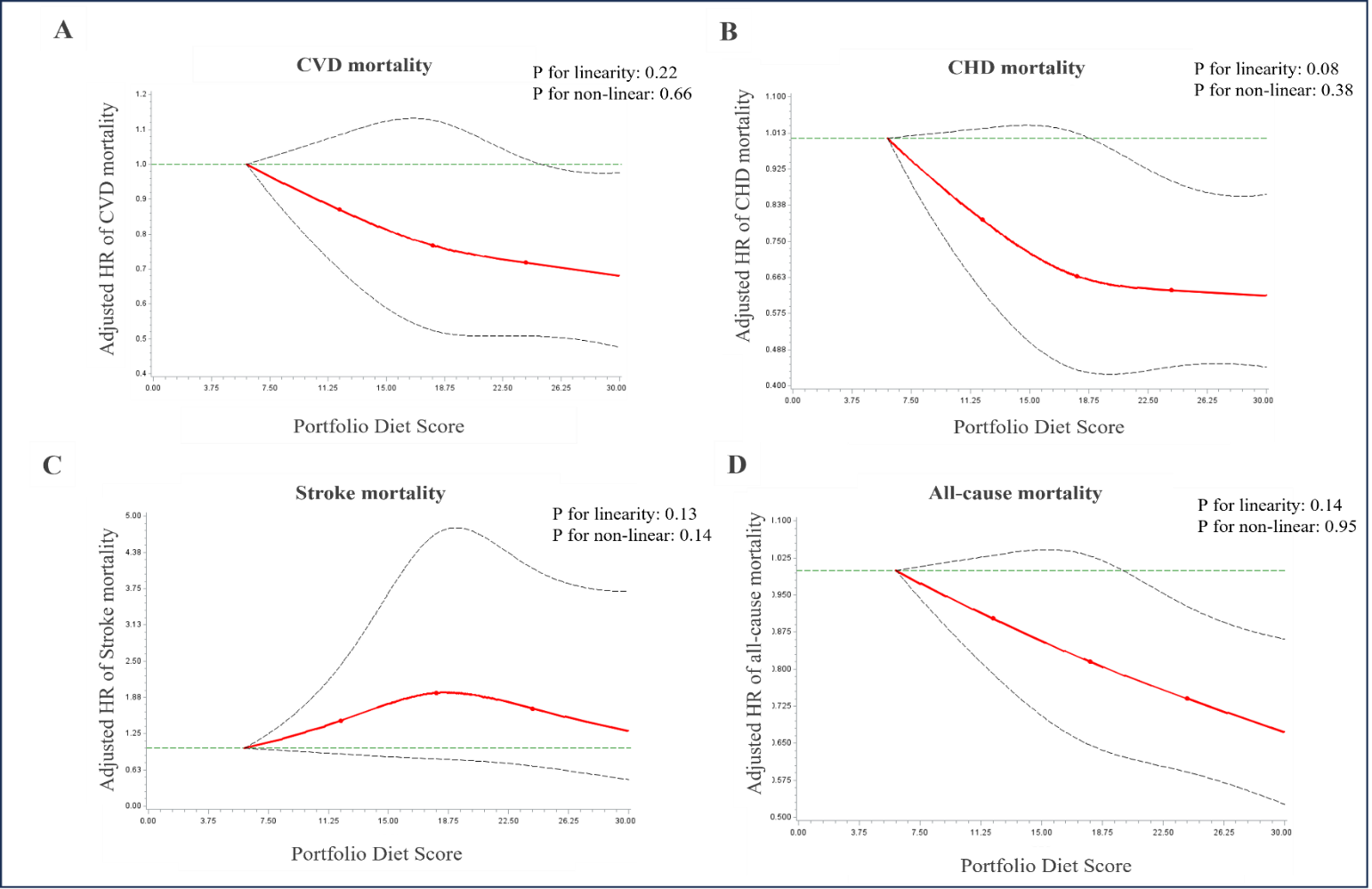


# **Figure S4.** Adjusted differences in hazard ratio for: (a) CVD mortality, (b) CHD mortality, (c) stroke mortality, and (d) all-cause mortality

Abbreviations: CI, confidence intervals; CVD, cardiovascular disease; CHD, coronary heart disease; HR, hazard ratio.

Cox regression models were used to estimate the adjusted differences in HR and corresponding 95% CI. HRs were coded using restricted cubic spline (RCS) function with three knots located at the 5^th^, 50^th^ and 95^th^ percentiles of the PDS distribution (reference value = 6, the lowest PDS value). Solid red line represents HR and dotted lines represent 95% CIs.

Estimates were adjusted by age, sex, and race/ethnicity, educational attainment, smoking status, PIR, marital status, physical activity, alcohol consumption, family history of CVD, self-reported cancer (other than skin), self-reported type-2 diabetes, self-reported hypertension, energy intake, and sodium.

CVD mortality defined as deaths identified as Disease of heart (ICD codes: I00-I09, I11, I13, I20-I51) and Cerebrovascular disease (ICD codes I60-I69). CHD mortality defined as deaths identified as Disease of heart (ICD codes: I00-I09, I11, I13, I20-I51). Stroke mortality defined as deaths identified as Cerebrovascular disease (ICD codes I60-I69)

# **Figure S5.** Subgroup analysis with adjusted hazard ratio for CVD mortality

Comparing Tertile 3 (highest) with Tertile 1 (lowest) of Portfolio Diet Score by Selected Characteristics Among US Adults 20 Years or Older: National Health and Nutrition Examination Survey (NHANES) Linked Mortality Files, 1988-2019. Red diamond represents adjusted HR and solid limit lines indicate 95% CIs. Vertical dotted line represents the adjusted HR for all participants.

Abbreviations: BMI, Body mass index; CI, confidence intervals; HR, Hazard Ratio; PIR, Poverty Income Ratio; T2D, Type-2 diabetes.

*P* value is for interaction across subgroups for all tertiles

*White includes participants with reported non-Hispanic White race/ethnicity

^†^Non-White includes all other participants including those with reported non-Hispanic Black, Mexican American, and other race/ethnicity. The other race/ethnicity includes all other Hispanics regardless of race (such as Other Latin American/Spanish ancestry or national origin) and also all other non-Hispanic adults from racial groups other than White or Black (i.e., American Indian or Alaskan Native; Native Hawaiian, Asia, or Pacific Islander; multiple races or ethnicities; or unknown). Consolidated owing to small number of events.

^‡^Physical activity was defined according to times/week. High physical activity included the participants who had moderate intensity to vigorous activities 5 or more times per week, whereas low physical activity included the participants who had none or moderate intensity to vigorous activities less than 5 times per week.

^§^More than 5% with missing values (hypercholesteremia, *n*=7,104).

Type 2 diabetes, hypertension and hypercholesterolemia were self-reported.

**Figure S6.** Subgroup analysis with adjusted hazard ratio for CHD mortality

Comparing Tertile 3 (highest) with Tertile 1 (lowest) of Portfolio Diet Score by Selected Characteristics Among US Adults 20 Years or Older: National Health and Nutrition Examination Survey (NHANES) Linked Mortality Files, 1988-2019. Red diamond represents adjusted HR and solid limit lines indicate 95% CIs. Vertical dotted line represents the adjusted HR for all participants.

Abbreviations: BMI, Body mass index; CI, confidence intervals; HR, Hazard Ratio; PIR, Poverty Income Ratio; T2D, Type-2 diabetes.

*P* value is for interaction across subgroups for all tertiles

*White includes participants with reported non-Hispanic White race/ethnicity

^†^Non-White includes all other participants including those with reported non-Hispanic Black, Mexican American, and other race/ethnicity. The other race/ethnicity includes all other Hispanics regardless of race (such as Other Latin American/Spanish ancestry or national origin) and also all other non-Hispanic adults from racial groups other than White or Black (i.e., American Indian or Alaskan Native; Native Hawaiian, Asia, or Pacific Islander; multiple races or ethnicities; or unknown). Consolidated owing to small number of events.

^‡^Physical activity was defined according to times/week. High physical activity included the participants who had moderate intensity to vigorous activities 5 or more times per week, whereas low physical activity included the participants who had none or moderate intensity to vigorous activities less than 5 times per week.

^§^More than 5% with missing values (hypercholesteremia, *n*=7,104).

Type 2 diabetes, hypertension and hypercholesterolemia were self-reported.

**Figure S7.** Subgroup analysis with adjusted hazard ratio for stroke mortality

Comparing Tertile 3 (highest) with Tertile 1 (lowest) of Portfolio Diet Score by Selected Characteristics Among US Adults 20 Years or Older: National Health and Nutrition Examination Survey (NHANES) Linked Mortality Files, 1988-2019. Red diamond represents adjusted HR and solid limit lines indicate 95% CIs. Vertical dotted line represents the adjusted HR for all participants.

Abbreviations: BMI, Body mass index; CI, confidence intervals; HR, Hazard Ratio; PIR, Poverty Income Ratio; T2D, Type-2 diabetes.

*P* value is for interaction across subgroups for all tertiles

*White includes participants with reported non-Hispanic White race/ethnicity

^†^Non-White includes all other participants including those with reported non-Hispanic Black, Mexican American, and other race/ethnicity. The other race/ethnicity includes all other Hispanics regardless of race (such as Other Latin American/Spanish ancestry or national origin) and also all other non-Hispanic adults from racial groups other than White or Black (i.e., American Indian or Alaskan Native; Native Hawaiian, Asia, or Pacific Islander; multiple races or ethnicities; or unknown). Consolidated owing to small number of events.

^‡^Physical activity was defined according to times/week. High physical activity included the participants who had moderate intensity to vigorous activities 5 or more times per week, whereas low physical activity included the participants who had none or moderate intensity to vigorous activities less than 5 times per week.

^§^More than 5% with missing values (hypercholesteremia, *n*=7,104).

Type 2 diabetes, hypertension and hypercholesterolemia were self-reported.

**Figure S8.** Subgroup analysis with adjusted hazard ratio for all-cause mortality

Comparing Tertile 3 (highest) with Tertile 1 (lowest) of Portfolio Diet Score by Selected Characteristics Among US Adults 20 Years or Older: National Health and Nutrition Examination Survey (NHANES) Linked Mortality Files, 1988-2019. Red diamonds represent adjusted HR and solid limit lines indicate 95% CIs. Vertical dotted line represents the adjusted HR for all participants.

Abbreviations: BMI, Body mass index; CI, confidence intervals; HR, Hazard Ratio; PIR, Poverty Income Ratio; T2D, Type-2 diabetes.

*P* value is for interaction across subgroups for all tertiles.

^*^White includes participants with reported non-Hispanic White race/ethnicity

^†^Non-White includes all other participants including those with reported non-Hispanic Black, Mexican American, and other race/ethnicity. The other race/ethnicity includes all other Hispanic adults regardless of race (such as Other Latin American/Spanish ancestry or national origin) and also all other non-Hispanics from racial groups other than White or Black (i.e., American Indian or Alaskan Native; Native Hawaiian, Asia, or Pacific Islander; multiple races or ethnicities; or unknown). Consolidated owing to small number of events.

^‡^Physical activity was defined according to times/week. High physical activity included the participants who had moderate intensity to vigorous activities 5 or more times per week, whereas low physical activity included the participants who had none or moderate intensity to vigorous activities less than 5 times per week.

^§^More than 5% with missing values (hypercholesteremia, *n*=7,104).

Type 2 diabetes, hypertension and hypercholesterolemia were self-reported.
